# Systematic Analysis of the Literature in Search of Defining Systemic Sclerosis Subsets

**DOI:** 10.3899/jrheum.201594

**Published:** 2021-05-15

**Authors:** Tatiana Nevskaya, Janet E. Pope, Matthew A. Turk, Jenny Shu, April Marquardt, Frank van den Hoogen, Dinesh Khanna, Jaap Fransen, Marco Matucci-Cerinic, Murray Baron, Christopher P. Denton, Sindhu R. Johnson

**Affiliations:** 1 Schulich School of Medicine & Dentistry, Western University, London, Ontario, Canada; 2 University of Michigan, Ann Arbor, Michigan, USA; 3 St. Maartenskliniek and Radboud University Nijmegen Medical Centre, Nijmegen, the Netherlands; 4 Radboud University Nijmegen Medical Centre, Nijmegen, the Netherlands; 5 Department of Experimental and Clinical Medicine & Division of Rheumatology AOUC, Florence Italy University of Florence, Florence, Italy; 6 McGill University, Division Head Rheumatology, Jewish General Hospital, Montreal, Quebec, Canada; 7 University College London, Division of Medicine, London, UK; 8 Toronto Scleroderma Program, Toronto Western and Mount Sinai Hospitals, Department of Medicine, and Institute of Health Policy, Management and Evaluation, University of Toronto, Toronto, Ontario, Canada.

**Keywords:** autoimmune diseases, scleroderma, systemic sclerosis

## Abstract

**Objective.:**

Systemic sclerosis (SSc) is a multisystem disease with heterogeneity in presentation and prognosis. An international collaboration to develop new SSc subset criteria is underway. Our objectives were to identify systems of SSc subset classification and synthesize novel concepts to inform development of new criteria.

**Methods.:**

Medline, Cochrane MEDLINE, the Cumulative Index to Nursing and Allied Health Literature, EMBASE, and Web of Science were searched from their inceptions to December 2019 for studies related to SSc subclassification, limited to humans and without language or sample size restrictions.

**Results.:**

Of 5686 citations, 102 studies reported original data on SSc subsets. Subset classification systems relied on extent of skin involvement and/or SSc-specific autoantibodies (n = 61), nailfold capillary patterns (n = 29), and molecular, genomic, and cellular patterns (n = 12). While some systems of subset classification confer prognostic value for clinical phenotype, severity, and mortality, only subsetting by gene expression signatures in tissue samples has been associated with response to therapy.

**Conclusion.:**

Subsetting on extent of skin involvement remains important. Novel disease attributes including SSc-specific autoantibodies, nailfold capillary patterns, and tissue gene expression signatures have been proposed as innovative means of SSc subsetting.

Systemic sclerosis (SSc) is a multisystem autoimmune rheumatic disease characterized by microvascular injury and accumulation of collagen in skin and other organs, such as the musculoskeletal system, lungs, kidneys, and gastrointestinal (GI) tract.^1,2,3,4,5,6^ SSc is associated with poorer patient outcomes and lower quality of life when compared to other rheumatic diseases.^7^ The 2013 American College of Rheumatology/European League Against Rheumatism (ACR/EULAR) classification criteria for SSc include skin thickening, fingertip lesions, abnormal nailfold capillaries, and the presence of SSc-related autoantibodies, but do not differentiate subsets of patients with SSc.^8^ Subclassification of SSc into a number of pathogenetically homogenous subsets with similar clinical manifestations and outcomes would help segregate clearly between prognostically distinct disease subgroups. Despite the complex multiorgan nature of SSc, the subsets are frequently defined as being limited cutaneous (lcSSc) or diffuse cutaneous (dcSSc), based on the location of skin involvement.^9^ This classification system gives insight into disease progression; however, within lcSSc and dcSSc, the course of disease is highly variable between patients.^10,11^ With a more modern perspective, our understanding of SSc subsets is changing. A combination of multisystem involvement, antibody profiling, genetic markers, and differences in proteomics may play a role in prognosis and treatment options.^12,13,14,15,16^ Further defining subsets of patients with SSc may help to prognosticate, especially in early disease.^17^

An international collaboration to develop new criteria to subset SSc is underway.^18^ Current perceptions around SSc subset criteria were identified by leading international experts. In a survey of 30 SSc experts from 13 countries, 90% of experts use > 2 subsets for classifying and treating their patients.^19^ Concepts such as progression rates and likely organ involvement are considered for subsetting patients with SSc informally in clinical practice.

There is a need for criteria to identify subsets of patients with SSc for recruitment into clinical trials of novel therapeutic agents, to inform management, and for prognosis in clinical care. Previous attempts to outline SSc subset classification criteria have relied mainly on clinical manifestations.^20^ However, in recent years, novel disease attributes including autoantibody profiles, nailfold capillary patterns, and gene expression signatures have been proposed as means of subsetting. The objectives of this study were to identify existing systems of subset classification in SSc and to synthesize novel concepts in subsetting through a systematic review of the literature.

## METHODS

### Data sources and search strategy.

A search of publications related to SSc and subsets was performed using Medline, Cochrane MEDLINE, the Cumulative Index to Nursing and Allied Health Literature, EMBASE, and Web of Science from their inceptions to December 2019 (for search strategy and key terms, see Supplementary Table 1, available with the online version of this article). The research question was, “What are the advantages and disadvantages of existing systems of subset classification in patients with systemic sclerosis?”

Searches were supplemented by hand searching the bibliographies of relevant articles (including citation searching). Studies were limited to humans, without language or sample size restrictions. Non–English-language articles were translated by native-language speakers or machine software. EndNoteX9 software (Clarivate) was used to check for duplications.

Studies were screened and excluded if they (1) reported localized scleroderma or scleroderma-like syndromes; (2) were abstracts, case reports, or review articles; or (3) were studies for which updated manuscripts were available. All articles were divided between 4 research groups (DK/CD, JF/FV, MM/JP/JS/TN, MB/SJ/TN) and independently reviewed by investigators from each group using a standardized data abstraction form. Abstracted data included classification schema, number of SSc subsets, number of subjects, country of origin, stated and perceived advantages and disadvantages of the classification system, and external validation. The systematic review conforms to the Preferred Reporting Items for Systematic Reviews and Meta-Analyses statement. The Strengthening the Reporting of Observational Studies in Epidemiology (STROBE) checklist was used to assess the reporting quality of the included studies.

## RESULTS

### Search results.

**Figure 1.**
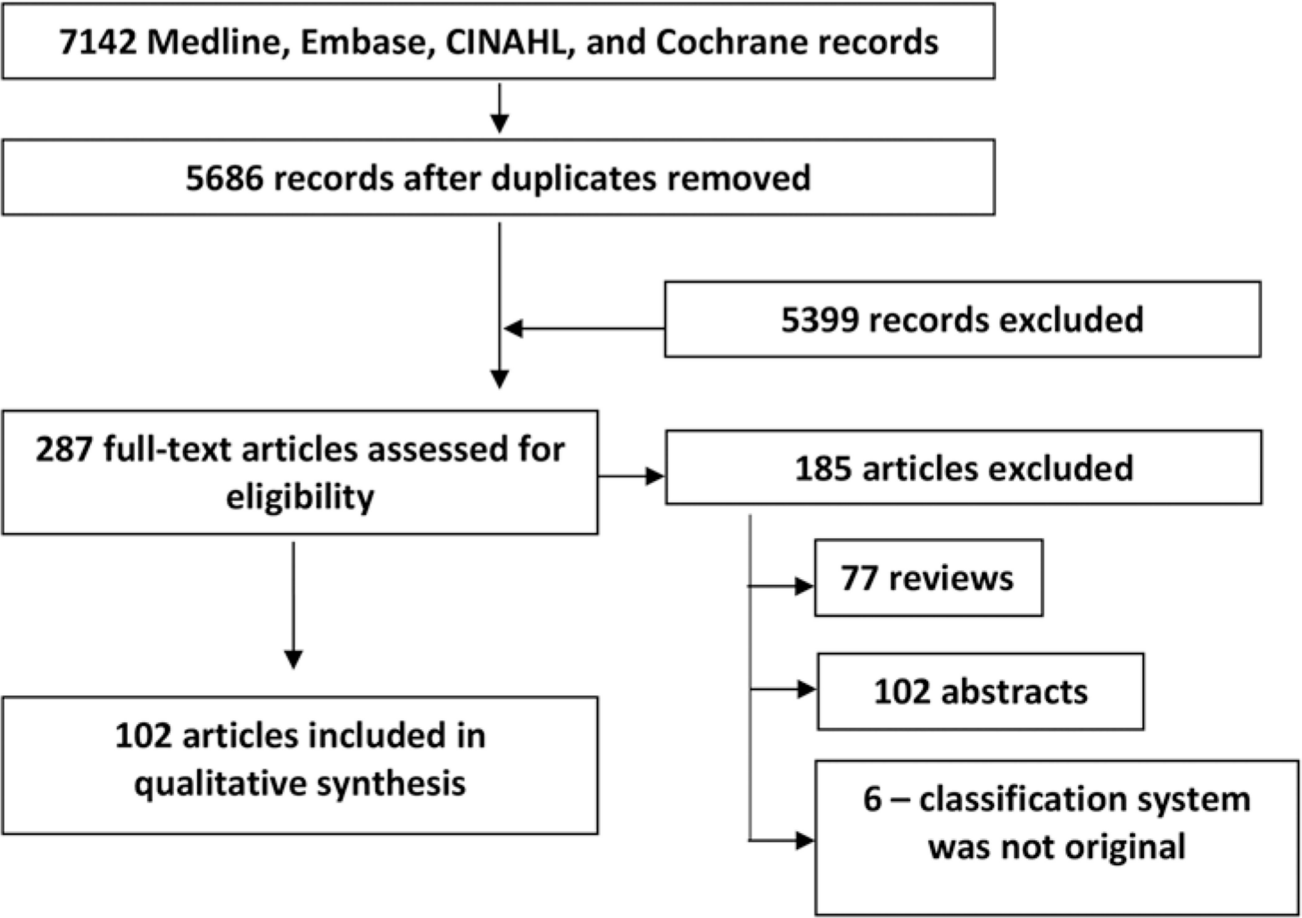
Flow diagram of search results. CINAHL: Cumulative Index to Nursing and Allied Health Literature.

Our literature review identified 5686 citations, of which 5584 were excluded because they were not relevant (conditions other than SSc, no classification system proposed), they had insufficient data, the data were not original, and/or they did not involve humans. The remaining 102 studies reported schema to subset patients with SSc (Figure 1).

### SSc subset criteria.

Subset classification systems have historically relied on clinical manifestations, most commonly extent of skin involvement (n = 20; Table 1),^9,10,11,21–37^ molecular, genomic, and cellular patterns (n = 12; Table 2),^38–49^ SSc-specific autoantibodies (n = 46, including 5 studies exploring both clinical and serological subsets^10,21,27,29,37^; Table 3),^10,21,27,29,37,50–90^ and abnormal nailfold capillary patterns (n = 10; Table 4).^91–100^ Twenty-one studies reporting associations between capillary abnormalities and clinical features or serology were included (Table 5).^94,99,101–119^ Using the STROBE checklist, the majority provided a clear presentation of what was planned, done, and found (Supplementary Table 2, available with the online version of this article).^120^

### SSc subsets based on the extent of skin involvement.

The diffuse vs limited SSc criteria of LeRoy, *et al*^9^ is the most commonly used system of SSc classification. The differences in development of visceral (renal and myocardial) disease and survival were shown for the subsets.^9,11,25,26^ The system has a good discriminative value to identify the groups of patients with different dominant features (vascular vs fibrotic), internal organ damage, and outcome. It enables identification of patients with early SSc with poor prognosis who will need close monitoring and facilitates the comparison of more homogenous groups of patients in epidemiological studies and clinical trials. The LeRoy 1988 classification system^9^ has the advantage of comprising only 2 groups and requires criteria other than cutaneous involvement. To classify as diffuse SSc (dSSc), the prerequisites are the onset of Raynaud phenomenon (RP) within 1 year of the onset of skin involvement, early and significant visceral involvement, and the absence of anticentromere antibodies (ACA). When using these strict LeRoy criteria, dSSc represents only a small portion (8.5%) of the total group with definite SSc.^23^ Two SSc-specific autoantibodies were included in the original LeRoy criteria: antitopoisomerase I antibodies (ATA) and ACA.

Acknowledging the important role of autoantibodies and capillary abnormalities, LeRoy updated the classification in 2001, proposing 4 subsets: limited SSc (lSSc), lcSSc, dcSSc, and diffuse fasciitis with eosinophilia. The classification includes lSSc as RP only in association with serological and/or capillary abnormalities.^32^ Considering that SSc is a multistage multiorgan disorder, lSSc is likely an early stage of disease and corresponds to very early SSc in the classification of Avouac, *et al*.^28^

Others have proposed 3 subset systems based on the extent of cutaneous involvement within the first year of presentation: type I digital (finger or toe skin involvement), type II intermediate (skin involvement proximal to metacarpophalangeal [MCP] joints, but excluding trunk), and type III diffuse (truncal sclerosis).^10,24,29,33^ The latter type was characterized by male predominance, shorter RP before skin changes, and worse prognosis.^11^ The clinical distinctiveness of the types was confirmed by difference in autoantibody profile: ACA was found more frequently in type I, while ATA was more frequent in intermediate SSc (iSSc) and dSSc. In the study, the authors included only SSc patients with disease duration ≤ 2 years after the onset of skin lesions, and none of the patients had received any treatment that could potentially affect skin sclerosis prior to the enrollment. That ruled out the possibility that the iSSc group consisted of patients with SSc that would evolve into dSSc later or who originally had dSSc with skin regression under the treatment. Compared to the 2-subset LeRoy system, this classification better reflects the clinical heterogeneity of disease and identifies the subgroups with milder or more severe clinical prognostic evolution.

The simplicity of this 3-subset classification, which is based on clinical examination of skin only and does not require special equipment or tests, makes it highly reproducible and suitable for clinical care and research studies. Notably, this classification system includes a time determinant reflective of the pace of disease, and thus has a prognostic value. Barnett, *et al*^10^ emphasized the importance of assessing the extent of skin involvement within the first year of presentation to place a patient into a specific type. Indeed, type I and II patients had a better prognosis in terms of life expectancy compared to type III. However, only slight difference in survival was found between patients with iSSc and those with lSSc.

Patients with iSSc were found to have variable clinical features and represented a serologically heterogeneous group. It raises the question of iSSc as a distinct variant. Some authors suggested that further subdivision of iSSc might be necessary to identify the subsets with particular patterns of internal organ damage and outcome. Scussel-Lonzetti, *et al*^25^ divided iSSc into “above elbow” and “below elbow” groups but found them similar with respect to internal organ involvement, mortality, and autoantibody profile. Although the authors supported the concept of an iSSc subset, differentiation was shown only between the LeRoy subsets (“normal + limited” vs “intermediate + diffuse”) in terms of heart involvement, disease activity (elevated erythrocyte sedimentation rate [ESR], anemia), and pulmonary fibrosis. The most significant difference in survival rates was found between lSSc and dSSc, whereas the difference between other subsets was absent (lSSc vs iSSc, *P* = 0.2) or very low (iSSc vs dSSc, *P* = 0.03). ATA positivity was similar between iSSc and dSSc while ACA frequencies gradually decreased from lSSc through iSSc to dSSc (50%, 34%, and 3.4%, respectively). Supporting the LeRoy system, the skin involvement proximal to MCP joints was one of the strong predictors of mortality. In line with those findings, Vayssairat, *et al*^23^ showed the advantages of LeRoy subset system and disutility of adding iSSc as a subset. When patients with proximal skin thickening were divided into intermediate and truncal subsets, no difference in severity score was found between them.

The patients with calcinosis, RP, esophageal involvement, sclerodactyly, telangiectasia (CREST) syndrome, suspected secondary RP, and/or visceral SSc without skin involvement were not acknowledged in the aforementioned 2 classification systems.^9,10^ The recently developed immunoblotting technique to detect SSc-related autoantibodies and nailfold capillary microscopy allows the detection of these probable connective tissue diseases. Expanding the subsets, Maricq, *et al*^22^ added undifferentiated connective tissue disorder with SSc features, SSc sine scleroderma, and CREST. This classification allows the inclusion of patients who are in earlier stages of their disease.

Boonstra, *et al*^27^ identified 4 clinical subgroups by hierarchical clustering using skin, musculoskeletal, cardiac, pulmonary, and GI manifestations; demographics; and risk assessment using follow-up data. Subgrouping patients allowed the prediction of severity and mortality with 2 subgroups showing higher-than-average 5-year mortality rates: subgroup 1 (male predominance, dcSSc, higher modified Rodnan skin score [mRSS], scleroderma renal crisis (SRC), ATA, less frequent interstitial lung disease [ILD]); and subgroup 2 (female and non-White predominance, more frequent pulmonary arterial hypertension [PAH], gastric antral vascular ectasia [GAVE], ILD, and lower diffusing lung capacity for carbon monoxide [DLCO] and forced vital capacity [FVC]). Low-risk clusters (subgroups 3 and 4) included patients with lcSSc who were predominantly female, had more frequent GI manifestations (dysphagia, diarrhea, constipation) for both subgroups, as well as peripheral vascular involvement (digital ulcers), ACA, and White predominance for subgroup 3, and less frequent ILD, FVC, and DLCO for subgroup 4. Three subgroups (1, 3, and 4) were similar to the clusters (6, 3, and 1, respectively) in another subclassification system developed by Sobanski, *et al* as a European Scleroderma Trials and Research Group clustering initiative.^37^ However, 2 main clusters, A and B, in the latter study strongly support the LeRoy 2001^32^ subclassification into dcSSc and lcSSc.

### SSc subsets based on molecular gene expression profiling.

Another approach to classifying patients with SSc into subsets is molecular phenotyping identified through gene expression profiling in tissue samples. Four subsets characterized by distinct molecular pathway signatures have been described and validated in multiple studies: fibroproliferative, inflammatory, normal-like, and limited.^38–45,49,121^ The intrinsic molecular subsets are consistent for each patient, as well as across the different skin biopsy sites, regardless of clinically affected or unaffected status.^38,122^ The subsets are also consistent across the organ systems^38,39,42,122^; however, highly lung-specific innate immune and cell proliferation processes were shown within the immune-fibrotic axis, suggesting that there are gene pairs that are more likely to interact in one tissue than the other (Table 2).^123^

### SSc subsets according to SSc-related autoantibodies.

The classification system according to serum antibodies is based on the findings of mutually exclusive, SSc-specific autoantibodies that did not change during the course of disease. The autoantibody subsets are distinguished by patterns of cutaneous involvement, specific clinical features, and prognosis (Table 3). SSc-specific autoantibodies were found to be stronger predictors of disease outcome and organ involvement than the extent of skin involvement.^27^ The subset of patients with SSc positive for ACA represents a clinically homogenous group with distinct clinical features and seems to have a better prognosis: less severity; less frequent ILD, SRC, inflammatory arthritis, and inflammatory myositis; and patients had lower rates of GI tract involvement, finger ulcers, digital tuft resorption, or finger contractions. The patients are also older at disease onset, predominantly female, and more likely to have limited disease, lower skin scores, telangiectasia and pulmonary hypertension.^10,21,29,51–57,59,61–63, 65,69–71,73,74,84,86,89,124^ ACA status was found to be predictive of the extent of skin involvement over time.^59^ Patients with limited disease who were ACA-negative at baseline were more likely to progress to diffuse disease. ACA-negative patients also had a greater extent of cutaneous involvement, worse survival, and more severe internal organ involvement.^29,65^

Another study supported subdivision of lcSSc into 2 serological subtypes, Th/To-positive and ACA-positive, with different internal organ involvement and outcome.^50^ Compared to the ACA-positive patients, Th/To-positive patients were younger at disease onset and predominantly male, with less PAH development, but more ILD (38% vs 4.5%). The highest mortality was found in ATA+ and ATA+/ACA– subgroups, while ACA+/ ATA– and Pm/Scl+/RNA polymerase antibody (RNAP)-negative patients were classified as low risk.^26^ Some patients were not within described serological subsets; for example, ACA was commonly found in association with mild skin involvement, but 9% of dcSSc patients with truncal involvement were positive for ACA.^10^

Caetano, *et al* described those patients who had a more insidious onset of skin and major organ involvement, a lower incidence of ILD and SRC, and better survival than expected for dcSSc as a distinct clinical subtype (dcSSc ACA+).^70^ Thus, further subgrouping within each autoantibody profile may be promising from a clinical point of view. Indeed, 2 subgroups of anti-CENPA can explain variable clinical manifestations in an ACA-positive subset.^87^ Subgrouping among patients with SSc positive for anti-RPC155 antibodies (RNAP III large subunit, 155 kDa) revealed that anti-RPA194 was associated with a lower cancer risk and less severe GI disease, while anti-RNAP I/II/III was associated with SRC.^75^ Therefore, different autoantibody combinations have utility as tools for organ involvement and cancer risk stratification in SSc.

Patterson, *et al*^86^ reported subgrouping RNAP III–positive patients into 2 clusters; a strongly positive cluster was associated with an increased risk of GAVE, lower risk of esophageal dysmotility, and shorter disease duration. A strong positivity for anti-RNAP III (a higher ELISA index) was associated with the development of SRC.^75^ Although 3 main autoantibodies (ACA, ATA, and anti-RNAP III) have strong mutually exclusive relationships, coexpression of other antibodies are relatively common.^86,90,125,126^ A combination of 2 SSc-related autoantibodies was revealed in one-third of patients in the study by Patterson, *et al*.^86^ Anti-Ro52 most frequently occurred in combination with other autoantibodies, but coexpressions of ATA with anti-RNAP III (0.6%) and ACA (3%) were also found in a small proportion of patients with SSc.^86^ In cases with coexistence of ≥ 2 autoantibodies, the autoantibody of highest titer determined the clinical phenotype.

### SSc subsets according to nailfold capillary abnormalities.

Capillary abnormalities seen on nailfold video capillaroscopy (NVC) can be used to subgroup SSc patients with different clinical manifestations and prognoses. There are 2 classification systems based on the NVC changes (Table 4). First, Maricq, *et al*^127^ described 2 capillary patterns: “slow” and “active.” Slow pattern was characterized by capillary telangiectasias and high number of extremely large (giant) capillary loops with a relatively well-preserved capillary distribution. The main feature of active pattern was moderate-to-extensive capillary loss associated with considerable distortion of the nailfold capillary bed and new blood vessel formation (bushy capillaries). Associations between capillaroscopic findings and disease activity, degree of progression, and prognosis were found. SSc patients with slow pattern predominantly had slowly progressive disease (new symptoms/signs during follow-up were found only in 1/11 patients), longer RP prior to entry, and were ACA-positive, while all patients with active pattern were ACA-negative and half showed disease progression. Capillary loss (active pattern) reflected disease progression that was confirmed in other publications.^98,114^ Ostojic, *et al*^103^ found that enlarged capillaries without a significant capillary loss (slow pattern) were more frequently seen in lcSSc, whereas giant capillaries (GCs) with advanced capillary loss (active pattern) occurred in dcSSc.

The Maricq NVC classification system has been further subdivided within the active pattern into “active” and “late,” whereas slow pattern was renamed as “early” by Cutolo, *et al.*^95,128^ The principal change was the interpretation of patterns as consecutive phases of progressive obliterative microangiopathy.^128^ Early pattern is characterized by a relatively well-preserved capillary distribution and density with a few enlarged capillaries/GCs, few capillary microhemorrhages, and no evident loss of capillaries. The following moderate loss of capillaries is a sign of the next active phase, with a mildly disturbed architecture of capillaries, frequent GCs and microhemorrhages, capillary derangement, and absent or few ramified capillaries (neoangiogenesis). The capillary changes typical for this phase (hemorrhages and GCs) are closely associated with disease activity. Sambataro, *et al* showed that NEMO score (cumulative number of microhemorrhages and microthrombosis) ≥ 6 was the best predictor of disease activity, followed by the GC score (number of GCs) ≥ 3.^118^ The active pattern had more severe disease manifested as extensive skin involvement and greater visceral involvement (muscle, kidney), and patients were ACA-negative in comparison with the early pattern.^91^ In the most advanced phase of SSc microangiopathy, represented by the late NVC pattern, the disorganization of the normal capillary array is generally seen, with severe loss of capillaries and large avascular areas, irregular enlargement of the capillaries, few or absent GCs, microhemorrhages, and ramified/bushy capillaries. Normal NVC pattern is rarely seen in SSc (4–12%), nearly exclusively in the lcSSc subset.^103,129^ Numerous studies confirmed that patients with more advanced NVC patterns had more severe disease.^91,92,93,98,103,127,129^ Significant capillary loss was more common in patients with lcSSc who met ACR criteria compared to those who did not.^115^

Classifying patients with SSc according to NVC patterns may predict development of a new organ involvement within 1 year.^98,100^ In 2 studies,^98,100^ the odds ratio to develop severe organ involvement (defined as a category 2 or higher in any of the 9 organ systems assessed according to the Medsger Disease Severity Scale, or new PAH or ILD at 18–24 months’ follow-up) was stronger according to more severe NVC patterns, adjusting for disease duration, subset, and vasoactive medications. These findings were externally validated in an Italian cohort.^100^ Associations between certain manifestations and NVC patterns are controversial, such as reduced capillary density and PAH.^107,108^ Sample size was sometimes too small to detect possible associations.^104^

All 3 NVC patterns can be observed in both clinical disease subsets (lcSSc and dcSSc)^128^; however, early pattern is more common in lcSSc, especially early lcSSc,^93^ whereas the late pattern is more prevalent in dcSSc.^92,93^ Classifying patients into NVC subsets is important early in the disease course because capillary loss is a reliable indicator of rapidly progressive early disease.^25,94^ Shenavandeh, *et al* showed that late pattern in patients with early SSc was associated with severity of finger contractures and significantly reduced pulmonary function, compared to active and early patterns.^94^ Table 4 demonstrates that the reduced number of capillaries typical for active and late patterns was more commonly seen in patients with longer disease duration, higher mRSS, more severe lung (including PAH), GI, and peripheral vascular involvement, a higher number of organs affected, and elevated ESR and C-reactive protein.^94,101–103,105,107,109–114,117,118,119^ The ACR criteria sensitivity may be improved by adding the NVC patterns.^115,116^ More severe NVC patterns (active and late) occurred in patients seropositive for ATA and anti-RNAP III, and negative for ACA.^93,95,117,119^ ANA-negative^99^ and ACA-positive^94^ patients had the most favorable early pattern. However, SSc-related autoantibodies are not directly linked with the development of a distinct SSc NVC pattern (Table 4 and Table 5).^129^

The limitations included small proportions of patients with each NVC pattern (especially early pattern), resulting in limited power to detect statistically significant differences. Some outcomes were omitted from the analysis (i.e., GI involvement and SRC), while others might have been interrelated (i.e., abnormalities in the cardiac measures might be secondary to pulmonary involvement, rather than present as primary cardiac involvement). Further, follow-up duration in the prospective studies varied and was relatively short. Definitions of organ involvement also varied between the studies, which made the comparison of the results difficult.

## DISCUSSION

SSc subset classification is a rapidly evolving field. Our systematic review highlights both the continued importance of skin involvement and the novel role of SSc-specific antibodies, abnormal nailfold capillary patterns, and molecular profiling in assessing patients to determine a subset.

The dcSSc subset comprises patients with rapidly progressive disease who require more aggressive treatment. However, disease progression assessed as severity-duration ratio (early significant visceral and skin involvement) suggests disease activity only in early dcSSc.^23,130,131^ In later stages of disease, patients classified as rapid progressors in the beginning may still have a high disease severity due to the accumulated significant damage, but low disease activity as a result of treatment or spontaneous remission. Some patients with SSc first develop severe skin involvement and/or visceral disease late in the disease course. Thus, the limited/diffuse system loses its predictive value in more advanced disease and should be supplemented with a necessary determination of disease activity and severity when it comes to choosing treatment. With the recent advances in antibody detection, some novel SSc-specific autoantibodies could be added to SSc subset classification autoantibody profiling to the skin involvement while determining a subset.

Based on gene expression profiling, patients with lcSSc can be assigned to the limited, inflammatory, or normal-like subsets, whereas fibroproliferative subset can be seen in patients with dcSSc. The molecular subsets seem to be a universal feature of SSc end-target organ pathology, not affected significantly by heterogeneity of skin involvement within a patient and/or fibroblast heterogeneity in tissues.^38,39,122^ The molecular intrinsic subset assignment could represent a valuable approach for matching patients with SSc to appropriate therapies. Molecular phenotyping may aid personalized medicine by identifying therapies with higher potential for success in each individual patient, as well as to select patients with SSc who will improve naturally as part of their disease course.^47^

Some limitations of subgrouping by molecular phenotyping include the relatively small sample sizes of clinical trials due to the rarity of disease itself, specific inclusion criteria that misrepresents the full spectrum of SSc, lack of controls, and differences in methods of transcript quantification and in the exact list of genes between studies. Moreover, not all therapy- or disease-relevant genes are regulated at the mRNA level. The use of molecular subsetting in clinical practice for individual patients is limited, as paired skin samples from each individual are often not available, analyses are not standardized, and large numbers of samples in a dataset are needed to identify the molecular subset with accuracy. Recently, supervised machine learning algorithms have been developed and may be successfully used to assign single samples to intrinsic gene expression subsets according to predefined criteria.^47^ The method utilizes a multinomial elastic net classifier and an optimized set of genes. Classifier accuracy in that study was proved using concordance of samples (83.3%) reporting Cohen κ coefficient (0.7391), and was externally validated. Further efforts are needed to explore molecular heterogeneity and intrinsic subsets in other tissues and particularly in peripheral blood, given its accessibility.

Attempts to identify SSc subsets considering SSc-specific autoantibodies have faced a variety of challenges. Boonstra, *et al* reported that adding autoantibody status to the cluster process resulted in correct classification of patients with ILD, PAH, and SRC.^27^ All high-risk patients were correctly identified by taking autoantibodies into account, but the number of patients incorrectly identified as possibly high risk increased significantly (by 66%), suggesting limited additional value of autoantibody status for clustering.^27^ The limitations of studies on SSc-specific autoantibodies included underestimation of the number of antigens due to the limitations of the techniques not allowing the identification of membrane proteins, or to a loss of proteins at each step, small sample size, a lack of validation groups, and/or limited generalizability (e.g., SRC is rare in Japanese patients; clinical features in each SSc-related ANA-based subgroup appear to vary among populations of different backgrounds). Feasibility is another consideration, as some autoantibodies are identified by immunoprecipitation, which is not widely used in clinical laboratories, and/or some detection kits are not commercially available. Limitations of classification systems developed by cluster analysis are the exclusion of a significant number of patients due to missing data and/or loss to follow-up that affects the extrapolation of the results. Finally, there have been inconsistent definitions of variables between the studies, a lack of analysis of the potential effect of treatment regimens on survival, and the influence of disease duration on the clustering process.

In conclusion, modern methods to subset SSc include skin involvement, immunologic profile, molecular signatures, visceral involvement, and age. Classifying on the basis of skin involvement, NVC, and autoantibody profile may allow early prediction of internal organ involvement. Molecular subsetting may identify those who are likely to respond to therapy. Longitudinal prospective studies to track subsets are needed to provide insight into disease trajectory, assess their predictive value, and confirm a possible transition between subsets and evolution under treatment.

## Supplementary Material

Appendix I

Appendix 2

## Figures and Tables

**Table 1.** Summary of clinical SSc subsets.

Citation	Country	STROBE	No. of Patients	List of Subsets

Ferri 1991^21^	Italy	18	150	Cutaneous: limited; intermediate; diffuse (higher % of men, worse prognosis, shorter RP before skin changes). Serological: ACA (higher % of female, lSSc, calcinosis, telangiectasia); ATA (iSSc and dSSc, GI and heart involvement, myositis, shorter RP duration before skin changes, skin ulcers, hyperpigmentation).
Ferri 2002^11^	Italy	17	1012	4 subsets: (1) sine scleroderma SSc: absence of cutaneous involvement with visceral involvement, nailfold capillary changes, and autoantibodies; (2) limited cutaneous: skin involvement of fingers with or without involvement of neck, face, and axillae; (3) intermediate cutaneous: skin involvement of upper and lower limbs, neck and face without truncal involvement; (4) diffuse cutaneous: distal, and truncal skin involvement.
Maricq 2004^22^	USA	18	165	(1) diffuse: skin involvement proximal to elbows/knees; includes trunk. (2) intermediate: skin involvement proximal to MCP/MTP, distal to elbows/knees; trunk not involved. (3) digital SD: sclerodactyly only; meets ACR minor criteria but excludes those without skin involvement. (4) SD sine SD: capillary pattern or pitting scars and visceral involvement; no ACA; no telangiectasia. (5) UCTD: 2/3 SD features (sclerodactyly, pitting scars, or SD capillary pattern), or 1/3 SD features and another 1 from an alternate group (RP, pulmonary fibrosis, or other visceral involvement [esophagus, heart, or kidney]), but do not meet the criteria of groups III and IV; those with CREST-type telangiectasia and/or ACA are excluded. (6) CREST: no skin involvement, or sclerodactyly only; telangiectasia is required with ≥ 1 other symptoms; or, ACA is required with any ≥ 2 symptoms.
Vayssairat 1992^23^	France	18	164	Comparison of different systems. (1) diffuse vs limited classification according to the criteria by LeRoy, *et al*^9^; (2) ARA classification: diffuse is defined as proximal to MCPs and distal is defined as a combination of ≥ 2 of the following—sclerodactyly (sclerodermatous involvement distal to the MCP), digital pitting scars, and bibasilar fibrosis as revealed by chest radiograph; (3) digital (finger or toe skin involvement), proximal extremity (proximal extremities but not trunk skin involvement), and truncal. Study included the accuracy of all these systems in reflecting disease severity (assessed by severity score).
LeRoy 1988^9^	USA	4	-	Two subsets: (1) dcSSc: onset of RP within 1 year; truncal and acral skin involvement; tendon friction rubs; early incidence of ILD, renal failure, diffuse GI disease, myocardial involvement; absence of ACA, abnormal NC. (2) lcSSc: RP for years; skin involvement limited to hands, face, feet, and forearms, or absent; late incidence of PAH, trigeminal neuralgia, calcinosis, telangiectasia; high incidence of ACA, abnormal NC.
Barnett 1969^24^	Australia	9	61	3 subsets: (1) limited, (2) moderate, and (3) extensive, based on skin involvement of the fingers only, limbs and face, and the trunk, respectively.
Barnett 1988^10^	Australia	10	177	Type 1: sclerodactyly only; Type 2: sclerosis proximal to MCP, but excluding trunk; Type 3: diffuse skin sclerosis, including trunk.
Scussel-Lonzetti 2002^25^	Canada	18	309	SSc without skin involvement, lSSc, iSSc, and dSSc. Further, iSSc was divided into “above and below elbow” forms.
Simeon 1997^26^	Spain	19	72	Group 1: sclerosis of fingers and neck; Group 2: sclerosis of face and distal to elbows; Group 3: generalized sclerosis, including trunk.
Boonstra 2018^27^	Netherlands	19	407	Clinical cluster analysis identified 4 subgroups, with 2 subgroups showing higher than average 5-year mortality rates. Adding autoantibody status to the cluster process resulted in 5 subgroups, with 3 showing higher than average mortality. High-risk subgroups: • Subgroup 1: male predominance, dcSSc, mRSS, SRC, ATA, less ILD; • Subgroup 2: female and non-White ethnicity predominance, PAH, GAVE, ILD, lower DLCO and FVC; • Subgroup 3: female and White ethnicity predominance, lcSSc, GI, reflux, constipation, diarrhea, peripheral vascular involvement (digital ulcers), ACA; • Subgroup 4: female predominance, lcSSc, GI, dysphagia, diarrhea, less ILD, FVC and DLCO.
Avouac 2011^28^	85 EUSTAR centers	19	-	Very early systemic sclerosis (VEDOSS: RP, puffy fingers, antinuclear antibodies, AND capillaroscopy OR SSc-specific antibodies
Giordano 1986^29^	Italy		90	Six subsets were studied. (1) sclerodactyly only; (2) sclerodactyly and skin involvement of neck, lower eyelid, or axillae; (3) skin involvement of hands and forearms ± legs ± face; (4) Group 3 and arm and/or thigh skin involvement; (5) Group 3 and thorax; (6) Group 3 and/or Group 4 and/or Group 5 and abdomen. Three subsets were designated: “limited” skin involvement of fingers, face, neck, axillae; “intermediate” skin involvement proximal to fingers; “diffuse” truncal skin involvement.
Goetz 1945^30^	USA	5	13	Two subsets: “acrosclerosis” and “diffuse”, based on skin thickening limited to extremities or includes trunk.
Holzmann 1987^31^	Germany	5	-	Five subsets (Types I-V) based on the extent and location of skin sclerosis, presence/absence of RP, extracutaneous manifestations, ANA
LeRoy 2001^32^	USA	5	-	Four subsets: (1) lSSc consists of (a) objective RP AND any 1 of NC changes or SSc selective autoantibodies OR (b) subjective RP AND both NC changes and SSc selective autoantibodies; (2) lcSSc criteria for lSSc plus distal cutaneous changes; (3) dcSSc^i^ criteria for lcSSc plus proximal cutaneous changes; (4) diffuse fasciitis with eosinophilia: proximal cutaneous changes without criteria for lSSc or lcSSc.
Masi 1988^33^	USA	6	-	Three subsets: digital - skin involvement of fingers or toes but not proximal extremity or trunk; proximal extremity - proximal extremities or face but not trunk; truncal - thorax or abdomen.
Rodnan 1979^34^	USA	6	273	Three subsets: (1) classical disease involving skin of the trunk, face, and proximal extremities, as well as early involvement of esophagus, intestine, heart, lung, and kidney; (2) CREST syndrome; and (3) overlap syndromes including sclerodermatomyositis and MCTD.
Winterbauer 1964^35^	USA	2	7	CRST syndrome: calcinosis, RP, sclerodactyly, telangiectasia.
Tuffanelli 1962^36^	USA	9	727	Two subsets: (1) acrosclerosis: RP, acral skin involvement; (2) dSSc: no RP, skin involvement beginning centrally.
Sobanski 20 19^37^	120 EUSTAR centers	19	6927	Two clusters: (1) lcSSc (81%), 2/3 without severe organ damage, ACA+ (54%); (2) dcSSc (61%), younger at disease onset, severe organ damage, ATA+ (54%), reduced survival.

ACA: anticentromere autoantibodies; ACR: American College of Rheumatology; ANA: antinuclear autoantibodies; ARA: American Rheumatism Association; ATA: antibodies to topoisomerase I; CREST: calcinosis, RP, esophageal involvement, sclerodactyly, telangiectasia; dcSSc: diffuse cutaneous SSc; DLCO: diffusing capacity for carbon monoxide; dSSc: diffuse SSc; GAVE: gastric antral vascular ectasia; EUSTAR: European Scleroderma Trials and Research; FVC: forced vital capacity; GI: gastrointestinal; ILD: interstitial lung disease; iSSc: intermediate SSc; lcSSc: limited cutaneous SSc; lSSc: limited SSc; MCP: metacarpophalangeal joints; MCTD: mixed connective tissue disease; mRSS: modified Rodnan skin score; MTP: metatarsophalangeal joints; NC: nailfold capillaroscopy; PAH: pulmonary arterial hypertension; RP: Raynaud phenomenon; SD: scleroderma; SRC: scleroderma renal crisis; SSc: systemic sclerosis; STROBE: Strengthening the Reporting of Observational Studies in Epidemiology checklist; UCTD: undifferentiated connective tissue disorder; VEDOSS: very early diagnosis Of SSc.

**Table 2.** Molecular, genomic, and cellular SSc subsets.

Citation	Country	STROBE	No. of Patients	List of Subsets

Milano 2008^38^	USA	21	24 SSc, 3 morphea, 6 healthy controls (skin)	• Normal-like, diffuse proliferation, inflammatory, limited signatures. • Diffuse proliferation: higher mRSS, all dcSSc, longer disease duration compared to patients with dcSSc in the inflammatory and normal-like groups; increased number of proliferating cells in the epidermis. • Inflammatory: both lcSSc and dcSSc; increased T cell infiltration in the dermis. • Limited: lcSSc, more severe RP. • Normal-like: both dcSSc and lcSSc.
Pendergrass 2012^39^	USA	17	22 dcSSc, 9 healthy controls (skin)	• Normal-like, fibroproliferative, inflammatory. • The gene-based subsets are reproducible, inherent, stable over time, and independent of disease duration. The intensity of the signature is associated with changes in disease duration and mRSS (i.e., high expression fibroproliferative subset associated with longer disease duration and higher mRSS; low expression inflammatory subset associated with higher mRSS). • No association with SSc-related autoantibodies.
Hinchcliff 2013^40^	USA	18	12 SSc, 10 healthy controls (skin)	• Normal-like, fibroproliferative, inflammatory. • Stable signatures over time, regardless of treatment; reproducibility; independence of autoantibody status; predicted response to MMF treatment: improvement mapped to inflammatory signature, while nonresponders belonged to normal-like and fibroproliferative subgroups.
Mahoney 2015 ^41^	USA	22	3 SSc patient cohorts from the studies^37,38,39^ (skin)	• Normal-like, fibroproliferative, inflammatory. • Identified the core sets of genes consistently associated with the intrinsic subsets, and created a gene-gene interaction network across the intrinsic subsets.
Taroni 20 1 5^42^	USA	21	16 SSc, 7 controls (esophageal biopsies)	• Inflammatory, noninflammatory, and proliferative. • Independent of dcSSc/lcSSc subtypes, serum autoantibodies, and esophagitis. • Inflammatory: older, a trend towards ILD (reduced DLCO, FVC, TLC).
Chakravarty 2015^43^	USA	22	13 SSc (10 treatment, 3 placebo), 4 healthy controls	• Fibroproliferative, inflammatory, and normal-like groups. • 4/5 improvers mapped to the inflammatory intrinsic subset showed decreased gene expression in inflammatory pathways over 24 weeks. One improver had normal-like signature (spontaneous improver?).
Gordon 2018^44^	USA	21	15 patients were assigned to either an inflammatory or a proliferative molecular subset at baseline	• Inflammatory, proliferative, normal-like. • Molecular subset at baseline was not associated with clinical improvement in the belimumab arm, the placebo arm, or the pooled treatment arms. • An overall reduction in inflammatory gene expression and movement toward the normal-like subset was associated with improvement in mRSS; 8/10 improvers were assigned to a normal-like molecular subset posttreatment.
Taroni 2017^45^	USA	16	Patients from multiple clinical trials	Immune and fibrotic signatures. High “inflammatory” signatures represented an active disease state. Epithelial-mesenchymal transition was significantly decreased in improvers from all trials. Different immunomodulatory treatments modulate distinct functional processes (i.e., ABA had higher scores for vascular- and collagen-related modules, while MMF had higher scores for proliferation and type I interferon modules).
Frost 2019^46^	South Africa, USA	15	8	Two groups co-segregated with clinical features of ILD and/or inflammatory myopathy, or the absence of an inflammation phenotype. These groups showed paradoxical gene expression of the genes *TCF7*, *SOX17*, and *FRZB* in affected and unaffected skin.
Franks 2019^47^	USA	21	297 skin biopsy samples from 102 patients with SSc and controls	Four intrinsic molecular subsets of SSc by supervised machine learning algorithms: fibroproliferative, inflammatory, normal-like, and limited.
van der Kroef 2020^48^	Netherlands, USA, Italy	19	19	Four clusters based on the distribution of monocyte subsets: • Cluster 1: high CD16+ monocytes and low memory B cell subsets, lcSSc; • Cluster 2: increased classical monocytes, dcSSc, high mRSS, the strongest increase of CXCL10 and CXCL11 in the plasma; • Cluster 3: larger amounts of memory B cells; • Cluster 4: lower numbers of circulating classical monocytes, often no skin involvement.
Martyanov 2017^49^	USA	20	19 patients with dcSSc (12 at baseline and posttreatment with dasatinib)	• Skin-based intrinsic gene expression: fibroproliferative, inflammatory and normal-like.

ABA: abatacept; dcSSc: diffuse cutaneous systemic sclerosis; DLCO: diffusing capacity for carbon monoxide; FVC: forced vital capacity; ILD: interstitial lung disease; lcSSc: limited cutaneous systemic sclerosis; MMF: mycophenolate mofetil; mRSS: modified Rodnan skin score; RP: Raynaud phenomenon; SSc: systemic sclerosis; STROBE: Strengthening the Reporting of Observational Studies in Epidemiology checklist; TLC: total lung capacity.

**Table 3.** Associations between SSc-related autoantibodies and clinical SSc manifestations.

Citation	Country	STROB	No. of Patients	Autoantibodies	Associations

Barnett 1988^10^	Australia	10	74	ACA	SSc type: a higher frequency of ACA in type 1 SSc sclerodactyly only (60.8%), followed by type 2 sclerosis proximal to MCP, but excluding trunk (29.7%), and type 3 diffuse skin sclerosis including trunk (9.5%).
Ceribelli 2010^50^	Italy, USA	18	216	anti-Th/To	• lcSSc and mild slowly progressive ILD • Compared to ACA+ subset, anti-Th/Th+ was associated with higher frequency of pericarditis, male sex, lower FVC, younger patients with SSc, and less frequent telangiectasia.
Gliddon 2011^51^	UK	15	180 lcSSc	ACA, ATA, anti-Th/To, anti-RNAP I/II/III, anti-Ul-RNP, unidentified ANA, ANA-negative	• ACA: older at disease onset, isolated reduction in DLCO, reduced creatinine clearance, telangiectasia, less frequent ILD • ATA: more extensive skin involvement, lung fibrosis • Anti-Ul-RNP: younger at disease onset, rare esophageal involvement, less frequent telangiectasia
Falkner 2000^52^	USA	19	282	ACA, ATA, anti-Th/To, anti-RNAP III, anti-fibrillarin, unidentified ANA	ACA and anti-Th/To — lcSSc
Graf 2012^53^	Australia	17	129 for clinical associations 298 for survival analysis	10 serological subtypes studied	dcSSc: • ATA: ILD, reduced survival • Anti-RNAP III: SRC, reduced survival lcSSc: • ACA: no ILD • Anti-Th/To: PAH • Anti-Ku: myositis (NS) Overlap: • Anti-Ul-RNP: frequent PAH, reduced survival, younger at disease onset • Anti-PM/Scl: ILD (NS)
Hamaguchi 2008^54^	Japan	20	203	ACA, ATA, anti-Ul-RNP, anti-RNAP; Anti-Th/To (small number of patients), inti-U3-RNP (small number of patients)	• ATA: dcSSc, high mRSS, diffuse skin hyperpigmentation, pulmonary fibrosis, decreased survival rate • Anti-RNAP: dcSSc, high mRSS, finger contractures • ACA: lcSSc, low mRSS, less frequent ILD • Anti-U3-RNP: dcSSc, rarely decreased DLCO • Anti-Ul-RNP: low mRSS • Anti-Th/To: low mRSS, rarely decreased DLCO and upper GI involvement • Negative ANA: low mRSS • dcSSc-positive for anti-RNAP (compared to dcSSc-positive for ATA): rapid skin progression, skin hyperpigmentation, less frequent pitting scars and ILD, lower serum IgG levels
Hanke 2010^55^ Ferri 1991^21^	Germany Italy	19 18	103 150	anti-CENP-A or anti-CENP-B ACA, ATA	• ACA (anti-CENP-A or anti-CENP-B): lSSc; less frequent ILD, cardiac involvement, skin ulcers • ACA: female predominance, lcSSc, calcinosis, telangiectasia • ATA: intermediate and diffuse SSc, GI and heart involvement, myositis, skin ulcers, hyperpigmentation, shorter RP duration before skin changes
Harvey 1999^56^ Hesselstrand 2003^57^	UK Denmark	19 19	155 276	ACA, ATA, anti-RNAP I/II/III ACA, ATA, anti-RNAP I/II/III, anti-Ul-RNP, antihistone	• ACA: lcSSc, rare renal disease and ILD • ATA: ILD, renal involvement (compared to ACA) • Anti-RNAP I/II/III: dcSSc • ACA: less frequent ILD, female predominance, vascular changes (finger systolic pressure), reduced GFR • ATA: dSSc, higher % of men, ILD • anti-RNAP I/II/III: ILD • anti-Ul-RNP: younger at disease onset, vasospasm • antihistone: more frequent cardiac, pulmonary and renal involvement, reduced survival
Song 2013^58^	China, USA	18	185	ACA* (anti-CENP-B and anti-CENP-Q)	less frequent ILD
Hudson 2012^59^	Canada	22	802	ACA	• ACA: older at disease onset, female predominance, lcSSc and lower mRSS, pulmonary hypertension, lower overall disease severity, less likely to have finger ulcers, digital tuft resorption, or finger contractures, ILD, SRC, inflammatory arthritis, and myositis. • ACA status was predictive of the extent of skin involvement over time. Patients with lcSSc who were CENP-A-negative at baseline were more likely to progress to diffuse disease.
Kuwana 2005^60^	Japan	20	534	Anti-RNAP III	dcSSc, higher maximum mRSS, and increased frequency of tendon friction rubs, SRC
McCarty 1983^61^	USA	17	27 ACA	ACA	Better prognosis, less frequent major renal, cardiac, pulmonary, and lower GI tract involvement compared to speckled or nucleolar ANA patterns
Vazquez-Abad I994^62^	USA	16	611	ACA (CENP-B)	CREST
Wu 2007^63^	Israel, USA	18	50 CREST 21 other	Anti-CCP3 in combination with ACA	CREST
Giordano 1986^29^	Italy	13	105	ACA	• ACA: sclerodactyly with/without minimal skin involvement in other areas (armpits, eyelids, neck) • ACA-negative (most were ATA-positive): arms, legs ± trunk involvement, lower cumulative survival rate and higher severity of internal organ involvement
Santiago 2007^64^	Canada	19	242	Anti-RNAP III	Risk of SRC
Salazar 2015^65^	USA	19	3249	ANA-negative	Less frequent vasculopathic manifestations
Satoh 2009^66^	Japan	18	354	Anti-RNAP III	Severe skin and renal involvement
Sato 1998^67^	Japan	20	103	anticalpastatin antibodies	Higher ESR and inflammatory muscle involvement
Simon 2009^68^	Hungary	19	293 (59 ATA positive)	ATA fragment F1	No clinical associations
Iniesta Arandia 2017^69^	Spain	19	209	ACA, ATA and anti-RNAP III-positive	• ACA: female predominance, less common dcSSc and ILD, longer time from onset to SSc diagnosis • ATA: higher prevalence of ILD, less frequent lcSSc and sine scleroderma subtypes • Anti-RNAP III: dcSSc, malignancies more frequent, especially synchronous neoplasia • No difference in terms of survival rate at 5 yrs and 30 yrs, or causes of death
Boonstra 2018^27^	Netherlands	19	407	5 clusters based on clinical and serological features	• Autoantibodies improved detection of lung involvement, PAH and renal crisis, as well as patients with actual severe disease course, when shifting from clinical subgrouping to combined autoantibody and clinical subgrouping. • High-risk (mortality around 10%): ∘ Subgroup 1: dcSSc and renal crisis, lower female predominance, ATA+ ∘ Subgroup 2: dcSSc, PAH, GAVE, less often White, ATA+, ACA- • Intermediate (mortality risk 7.2%): ∘ Subgroup 5: less frequent ILD and vasculopathy (pitting scars, digital ulcers), anti-RNAP III+, PM/Scl- • Low risk: ∘ Subgroup 3: GI, ACA+, ATA- ∘ Subgroup 4: miscellaneous, PM/Scl+, RNAP-
Caetano 2018^70^	UK	20	1313	ACA+ dcSSc, ACA+ lcSSc and ACA- dcSSc	dcSSc ACA+: insidious onset of skin and major organ involvement, a lower incidence of ILD and SRC, and better survival than expected for dcSSc
Caramaschi 2015 ^71^	Italy	5	178	ACA, ATA, anti-RNAP III, Th/To, PM/Scl	• ACA: older patients, longer disease duration from RP onset • ATA: ILD • anti-RNAP III: SRC
Coppo 2013^72^	France	19	199 individuals, including patients suffering from various autoimmune disorders (Group I, n = 145) and non autoimmune diseases (Group II, n = 44 patients) as well as healthy individuals (Group III, n = 30).	anti-HP1-positive	CREST
Igusa 2018^73^	USA	19	2383	ACA anti-RNAP III dcSSc and anti-RNAP lcSSc	• Anti-RNAP III+, ATA-, ACA-, anti-RNAP II; had increased risk of cancer • ACA+: lowest cancer risk • dcSSc anti-RNAP III: breast cancer • lcSSc anti-RNAP III: lung cancer
Foocharoen 2017^74^	Thailand	20	285	ATA, ACA (CENP A, CENP B), anti-PM/Scl-100, anti-PM/Scl-75, anti-Ku, anti-Ro52, anti-1RNAP III (RP11 and RP155), anti-fibrillarin (U3-RNP), anti-N OR-90, anti-Th/To, anti-PDGFR.	• ATA: female predominance, dcSSc, high peak mRSS, RP, hand deformity • ACA: negative association with hand deformity • Anti-Ku: overlap syndrome SSc/PM
Hamaguchi 2015^75^	Japan	20	583	Anti-RNAP III	Anti-RNAP III: SRC, in particular, coexistence of anti-RNAP II and anti-RNAP I/III (anti-RNAP I/II/III) and a higher ELISA index for anti-RNAP III
Haddon 2017^76^	USA	21	24	Anti–PM/Scl-100 as a part of the signature, also based on levels of CD40 ligand, chemokine (C-X-C motif) ligand 4 (CXCL4)	Clinical improvement
Foocharoen 2016^77^	Thailand	17	294	ATA, ACA	• ATA: hand deformity • ACA: negative association with hand deformity • ATA+dcSSc: earlier ILD vs ATA- • ATA-lcSSc: RP
Hoa 2016^78^	Canada, Australia, USA, Mexico	20	2140	anti-Ku	Anti-Ku: ILD, increased creatine kinase levels; no difference in survival
Terras 2016^79^	Germany	16	158(11)	Anti-RNAP III	dcSSc, higher mRSS, renal involvement
Perosa 2013^80^	Italy	21	121 (75 ACA positive)	ACA cross-reacting with FOXE3p53–62	Less likely to develop active disease
Wodkowski Canada, 2015^90^	Australia, USA	17	1574(103)	Monospecific anti-Ro52/TRIM21 antibodies	Less likely White, ILD, poor survival
Shah 2010^82^	USA	19	23(6)	anti-RNAP I/III	Temporal relationship with the onset of cancer
Sánchez-Montalvá 2014^83^	Spain	19	132	Anti-SSA/Ro52	No clinical associations
Shah 2019^84^	USA	18	168	anti-RPA194 (subgrouping among anti-RPC 155 antibodies)	Cancer, less severe GI disease
Shayakhmetova 201 9^85^	Russia	18	330 positive for a-U1RNP	anti-U1RNP	lSSc (91%), digital ulcers/scars (50%), ILD (63%); often joint (65%) and muscle (43%) involvement; 1/3 Sjogren syndrome
Patterson 2015^86^	Australia	18	505	ACA, anti-RNAP III (strong), anti-RNAP III (weak), ATA, anti-RNAP III, anti-N0R-90, anti-fibrillarin, anti-Th/To, anti-PM/Scl-75, anti-PM/Scl-100, anti-Ku, ATA, anti-Ro52, anti-PDGFR	• lSSc: ACA • dcSSc: RNAP III, ATA • Anti-Th/To: less likely joint contractures and reflux esophagitis • Anti-fibrillarin: digital amputation and a trend toward GAVE • Anti-TRIM-21/Ro 52: telangiectasia, dry eyes, PAH, and calcinosis • Anti-PM/Scl-75/100: a history of digital ulcers and a trend toward lcSSc, no history of smoking • Anti-RNAP III: dcSSc, joint contractures, SRC; a strong RNAP III cluster with increased risk of GAVE, lower risk of esophageal dysmotility, shorter disease duration
Perosa 2016^87^	Italy	21	84 anti-CENPA positive	Subspecificities of anti-CENPA: anti-pc4.2 antibodies, anti-pc14.1 antibodies	Anti-pc4.2 antibodies: sPAP and inversely associated with DLCO Anti-pc14.1 antibodies: inversely sPAP and positively DLCO
Wuttge 2015^88^	Denmark	19	95	ACA, ATA, anti-RNAP	Specific cell-free plasma miRNA profiles: • ACA: higher MiR-409–3p expression levels • ATA, anti-RNAPIII: higher MiR-184 • ATA, anti-RNP: lower MiR-92a
Wodkowski 2015^81^	Canada	17	16 monospecific anti PM75 and 11 anti-PM100	anti-PM75 and anti-PM100	• Both anti-PM75 and anti-PM100: myositis • anti-PM75: ILD, calcinosis • Anti-PM100: calcinosis, better survival
Liaskos 2017^89^	Greece, Germany, USA	19	131	ATA, ACA, a-RNAP III (RP11, RP155), anti-fibrillarin, anti-Ku, anti-N0R90, anti-PM- Scl100,anti-PM-Scl75	• ATA: dcSSc, ILD, PH and ILD-PH, digital ulcers (NS) • ACA (anti-CENPB): lcSSc, negatively ILD • anti-RP11: male sex • anti-NOR90: male predominance, ILD • anti-Ro52: arthritis
Sobanski 2019^37^	120 EUSTAR centers	19	6927	ATA, ACA	Six clusters (increasing mortality from 1 to 6): (1) lcSSc, predominately females, older at disease onset, GI involvement, low frequency of ILD, ACA (79%); (2) lcSSc, PH, ILD, ATA (35%), ACA (24%); (3) lcSSc, rare GI involvement and ILD, ACA (48%), ATA (24%); (4) lcSSc, severe cardiac, lung, GI, musculoskeletal, and peripheral vascular involvement; (5) dcSSc, predominately males, GI, cardiac, and lung involvement, ATA (50%), ACA (20%); (6) dcSSc, males, high peak mRSS, severe organ damage, ATA (77%), ACA (12%).

ACA: anticentromere autoantibodies; ANA: antinuclear autoantibodies; a-RNAP: antibodies to RNA polymerase; ATA: antibodies to topoisomerase I; CENP: centromeric protein; CREST: calcinosis, RP, esophageal involvement, sclerodactyly, telangiectasia; dcSSc: diffuse cutaneous SSc; DLCO: diffusing capacity for carbon monoxide; ESR: erythrocyte sedimentation rate; FVC: forced vital capacity; GAVE: gastric antral vascular ectasia; GFR: glomerular filtration rate; GI: gastrointestinal; ILD: interstitial lung disease; lcSSc: limited cutaneous SSc; MCP: metacarpophalangeal joints; mRSS: modified Rodnan skin score; NS: not significant; PAH: pulmonary arterial hypertension; PH: pulmonary hypertension; PDGFR: platelet-derived growth factor receptor; PM: polymyositis; RNAP: RNA polymerase antibodies; RP: Raynaud phenomenon; sPAP: systolic pulmonary artery pressure; SRC: scleroderma renal crisis; SSc: systemic sclerosis; STROBE: Strengthening the Reporting of Observational Studies in Epidemiology checklist.

**Table 4.** Associations between nailfold capillary patterns and clinical manifestations of SSc.

Citation	Country	STROBE	No. of Patients	Classification	Associations With Clinical Picture, SSc-related Autoantibodies, or Outcome

Chen 1984^91^	USA, China	18	68 SSc	Slow and active	• Slow capillary pattern: ACA • Active: extensive skin involvement and greater visceral involvement (muscle, kidney), more often hypertension
Caramaschi 2007^92^	Italy	21	103 SSc	Early, active, late	• Severity of skin, lung, heart, and peripheral vascular involvement, as well as homocysteine plasma levels progressively increased across the patterns, from early to late. • Early and active patterns were more common in lcSSc, whereas a late pattern was more common in dcSSc. • Late: increased risk of active disease, DUs and moderate-to-severe skin (mRSS ≥ 15), heart, and lung (lowest DLCO and FVC) involvement, risk of ILD
Ingegnoli 2013^93^	EUSTAR	21	2754 SSc	Early, active, late	Severity for skin involvement and number of systemic manifestations progressively increased across the patterns. • Early and active: mild/moderate skin involvement and a low number of disease manifestations • Late: more severe disease; ATA-positive cases with diffuse cutaneous involvement
Shenavandeh 2017^94^	Iran	19	70 SSc	Normal, early, active, late, nonspecific	• Early: early lcSSc (< 5 yrs) vs early dcSSc (> 3 yrs) • Late and active: skin telangiectasia, pitting scars, and pulmonary rales compared to those with early pattern • Late: limitation of the finger-to-palm range of motion, FEV1 < 70% compared to active and early (only in the early SSc subgroup and lcSSc subtype)
Cutolo 2004^95^	Italy	19	241 SSc	Early, active, late	Early and active: lcSSc, ACA+ Late: dcSSc, longer duration of RP and SSc, more advanced age, ACA-Active and late: ATA
Cutolo 20 16^96^	Europe, multicenter	22	623 SSc from 59 centers (14 countries)	Normal, early, active, late	Late: an increased risk of new digital ulcers during a 6-month observation period (OR for late vs normal/early pattern 4.2)
Bruni 2015^97^	Italy	17	110 SSc	Early, active, late	• Early and active: DUs (96%) compared to patients without a history or present DUs (66%) • Early: presence and/or history of DUs
Smith 20 12^98^	Italy	18	66 SSc	Normal, early, active, late.	The OR of future severe peripheral vascular and lung involvement at 18–24 months (defined as category 2–4 DSS per organ) rose steadily throughout the patterns.
Sulli 2013^99^	Belgium, Italy	15	42 SSc	Early, active, late	• ANA- patients had a slower progression of nailfold microangiopathy characterized by the early pattern. • Progression to the late pattern was associated with a different autoantibody pattern on IIF (fine-speckled + nucleolar pattern being most prevalent). • Late: ATA
Smith 2013^100^	Belgium, Italian	17	148	Normal, early, active, late	The OR to develop novel future severe organ involvement (in any of 9 organ systems, defined as category 2–4 per organ of the DSS at 18–24 months) was stronger according to more severe NVC patterns and similar in both cohorts.

ACA: anticentromere autoantibodies; ANA: antinuclear autoantibodies; ATA: antibodies to topoisomerase I; dcSSc: diffuse cutaneous SSc; DLCO: diffusing capacity for carbon monoxide; DSS: disease severity score; DU: digital ulcer; FEV1: forced expiratory volume in 1 second; FVC: forced vital capacity; IIF: indirect immunofluorescence; ILD: interstitial lung disease; lcSSc: limited cutaneous SSc; mRSS: modified Rodnan skin score; NVC: nailfold video capillaroscopy; RP: Raynaud phenomenon; SSc: systemic sclerosis; STROBE: Strengthening the Reporting of Observational Studies in Epidemiology checklist.

**Table 5.** Association between particular capillary abnormalities and clinical manifestations in patients with SSc.

Citation	Country	STROBE	No. of Patients	Classification	Associations With Clinical Picture, SSc-related Autoantibodies, or Outcome

Houtman 1985^101^	Netherlands	16	107: 39 isolated RP and 68 CTD (15 SSc, 9 CREST, 15MCTD)	Total no. of capillary loops, no. of enlarged capillaries	• Decreased number of capillary loops: sclerodactyly, DUs or pitting, tuft resorption, telangiectasia, the higher no. of organs affected, severe RP • Esophagus and lung involvement (radiograph), increased fibrinogen level and ESR • Increased no. of enlarged loops: lung involvement, arthralgia, elevated CRP • Decreased capillary density AND an increased number of enlarged loops: a positive Rose-Waaler test, latex agglutination test, ANA, and CIC
Bredemeier 2004^102^	Brazil	20	91 SSc	• Severity of capillary loss was evaluated on each digit as 0: no avascular areas; 1: 1 or 2 discrete areas of vascular deletion; 2: > 2 discrete areas of vascular deletion; 3: large confluent avascular areas. Severity scores ≥ 1 were considered severe capillaroscopic alterations. • MAS was calculated by dividing the sum of the scores by the number of digits examined • No. of megacapillaries	• MAS: higher mRSS, severity of sclerodactyly, signs of peripheral ischemia (pitting scars, finger amputation), esophageal dysfunction, ATA, ground-glass opacities, longer disease duration (a confounder due to end-organ damage) • Higher no. of megacapillaries per finger: ACA, ANA+ • Among patients with ≤ 5 yrs of disease duration, a greater no. of megacapillaries per finger was found in those with esophageal dysfunction; patients with ground-glass opacities had higher avascular scores and a tendency to a greater no. of megacapillaries per finger
Ostojic 2006^103^	Yugoslavia	16	105: 50 lcSSc, 55 dcSSc	Dilated capillaries without capillary loss; severe capillary damage/loss	• Enlarged capillaries without a significant capillary loss: lcSSc • Very enlarged capillaries with advanced capillary loss: dcSSc
Shenavandeh 2017^94^	Iran	19	70 SSc	GCs, capillary elongation, tortuosity, neoangiogenesis, reduced capillary density, avascular areas, abnormal blood flow and hemorrhages	• Neoangiogenesis, reduced capillary density, avascular area, and hemorrhages: limitation of the finger-to-palm range of motion • Neoangiogenesis: pitting scars • Avascular area: GI problems (any of dysphagia, heartburn, difficulty swallowing, the feeling of being full, vomiting, diarrhea, and constipation) • Giant loops: dysphagia • Abnormal blood flow: positive CRP • Capillary elongation: an inverse association with pitting scars • Capillary tortuosity: an inverse association with peripheral vascular manifestations
Lefford 1986^104^	UK	16	42 with CTD (14 RA, 19 SLE, 9 SSc)	Capillary parameters (apex, loop, and limb widths, loop length), no. of capillaries, interpeak capillary distance	• Greater apex, loop, and limb widths in SSc compared to controls and patients with R A • Shorter loop length and fewer capillaries, longer interpeak capillary distance, greater degree of variation in interpeak distances in SSc, compared to controls • No association with clinical manifestations and serological data
Lovy 1985^105^	USA	15	42	Capillary loss, capillary enlargement, telangiectasias	• Extreme capillary loss: longer disease duration • No significant correlation found between the presence or severity of capillary enlargement (and capillary loss) and the extent/ no. of organ involvement • Telangiectasias correlated with the presence and severity of nailfold capillary enlargement: all patients with extremely enlarged capillary loops had telangiectasias
Kenik 1981^100^	USA	14	24 with CTD (18 SSc)	Not detailed	No association between the degree of capillary changes and the stage of cutaneous disease
Hofstee 2009^107^	Netherlands	18	21 healthy controls, 20 idiopathic PAH, 40 SSc	Capillary density and loop dimensions	• Low capillary density: SSc-related PAH compared with those without PAH, while loop dimensions were equal • Capillary density: severity of PAH in both SSc-related and idiopathic PAH
Sato 2009^119^	Brazil	20	92 SSc	(1) no. of capillary loops/mm, (2) vascular deletion score assessed according to Lee method, (3) no. of enlarged loops, and (4) no. of giant capillary loops	Higher vascular deletion: mRSS, ATA+, finger pad lesions, ≥ 3 internal organs involved, dcSSc, compared to lcSSc, SSc sine scleroderma, and overlap syndrome
Greidinger 2001^108^	USA	20	37PPH, 15 SSc, 13 healthy controls	Capillary loop enlargement, dropout, density, bushy and tortuous capillaries	No difference between SSc patients with and without PAH
Alivernini 2009^109^	Italy	20	130 SSc	Avascular areas	Avascular areas: a major risk factor for the development of skin ulcers with a negative effect on healing
Sebastiani 2009^110^	Italy	16	120 SSc	Total no. of capillaries in the distal row (N), maximum loop diameter (D), number of megacapillaries (M), and the M:N ratio	The CSURI (D x M:N^2^) at the cutoff value of 2.94 represents a novel tool with the ability to predict the development of DUs in patients with SSc
Sebastiani 2012^111^	Italy	15	229 SSc	CSURI	High specificity (81.4%), sensitivity (92.98%) at the cut-off value of 2.96, and reproducibility (κ statistic measure of interrater agreement of 0.8514) of CSURI for the persistence and/or appearance of new DUs
Sebastiani 2013^112^	Italy	14	170 SSc	CSURI	CSURI showed good sensitivity, specificity, positive and negative predictive value
Manfredi 2015^113^	Italy	17	219 SSc	CSURI	• Altered CSURI is one of the factors associated with the appearance of DU • A prediction risk chart of the development of DUs within 6 months with 4 risk classes were built on the basis of CSURI, male sex, history of DUs, and ESR
Avouac 2017^114^	France, Italy	21	140 SSc	Number of capillaries, giant capillaries	• Increased number of giant capillaries: less risk to develop new DUs • Loss of capillaries within a follow-up: overall disease progression, appearance of new DUs, progression of pulmonary vascular involvement, skin fibrosis, and worsening of the Medsger severity score
Lonzetti 2001^115^	Canada	7	259 SSc	• Capillary dilatation (0 = normal; 1 = borderline [< 2 × normal diameter]; 2 = definitely dilated [≥ 2× but ≤ 4× normal diameter]; 3 = extremely dilated [> 4× normal diameter]) • Avascular areas (A: no capillary loss; B: rare avascular areas; C: moderate capillary loss; D: extensive capillary loss)	• Severe capillary loss (grade C or D avascular areas) : lcSSc ACR+ vs the lcSSc ACR— group. • The sensitivity of ACR criteria was improved from 33.4% to 74.3% by adding grade 2 or 3 dilated capillaries, then further to 82.9% by grade C or D avascular areas, and to 88.8% with clinically visible capillary telangiectasias
Hudson 2007^116^	Canada	18	101 SSc	Nailfold capillary abnormalities defined as the presence or absence of any dilated loops, GC loops and/or avascular areas for each digit; no scoring was done	The sensitivity of the ACR criteria in lcSSc was improved from 67% to 99% by adding nailfold capillary abnormalities and clinically visible telangiectasias
Herrick2010^117^	UK	18	176 SSc	Capillary width, distance between capillaries, density, tortuosity, and derangement	• Both automated and manually measured distance between capillaries: severe digital ischemia, ACA+ • Reduced density: ACA- • Wider capillaries: moderate/severe telangiectasias
Sulli 2013^99^	Belgium, Italy	15	42 SSc	No. of capillaries	A slight reduction of capillary number at baseline: either the nucleolar or the fine-speckled and nucleolar pattern on I IF
Sambataro 2014^118^	1Italy	19	107 SSc	• NEMO score (cumulative number of MHEs andMTs), • GC and C scores (total no. of G Cs and the mean no. of normal or slightly dilated capillaries)	• NEMO score: ESSG index scores, mRSS, scleredema, worsening of skin, cardiopulmonary, and vascular features, current DUs, and ESR > 30 mm/h • GC score: ESSG index score, mRSS, scleredema, DUs and worsening of cutaneous, vascular, and cardiopulmonary features • C score: negatively with ESSG index and mRSS, lower in patients with sclerodema, DUs, and DLCO < 80% • A NEMO score ≥ 6 is the best predictor of disease activity, followed by a GC score ≥ 3, and a C score ≤ 6, with the most balanced performance in terms of sensitivity/specificity ratio and the best accuracy

ACA: anticentromere autoantibodies; ACR: American College of Rheumatology; ANA: antinuclear autoantibodies; ATA: antibodies to topoisomerase I; C score: capillaries score; CIC: circulating immune complexes; CREST: calcinosis, RP, esophageal involvement, sclerodactyly, telangiectasia; CRP: C-reactive protein; CSURI: Capillaroscopic Skin Ulcer Risk Index; CTD: connective tissue disease; dcSSc: diffuse cutaneous SSc; DLCO: diffusing capacity for carbon monoxide; DU: digital ulcer; ESR: erythrocyte sedimentation rate; ESSG: European Scleroderma Study Group index; GC: giant capillaries; GI: gastrointestinal; IIF: indirect immunofluorescence; lcSSc: limited cutaneous SSc; MAS: mean avascular score; MCTD: mixed connective tissue disease; MHE: microhemorrhages; mRSS: modified Rodnan skin score; MT: microthrombosis; NEMO: cumulative number of MHEs and MTs; PAH: pulmonary arterial hypertension; PPH: primary pulmonary hypertension; RA: rheumatoid arthritis; RP: Raynaud phenomenon; SLE; systemic lupus erythematosus; SSc: systemic sclerosis; STROBE: Strengthening the Reporting of Observational Studies in Epidemiology checklist.
