# Supplementary material for: Systematic Analysis of the Literature in Search of Defining Systemic Sclerosis Subsets
Source: J Rheumatol. Author manuscript; Available in PMC 2023 Oct 29. (PMC10613330; doi:10.3899/jrheum.201594)
Supplement: Appendix I [file NIHMS1895227-supplement-Appendix_I.docx]

Appendix I. Reporting quality of the articles by using the STROBE checklist

| STROBE checklist | | | | | | | | | | | | | | | | | | | | | | | |
| --- | --- | --- | --- | --- | --- | --- | --- | --- | --- | --- | --- | --- | --- | --- | --- | --- | --- | --- | --- | --- | --- | --- | --- |
| First Author  Year | Total Score | Title, abstract, Introduction* | | | Methods* | | | | | | | | | Results* | | | | | Discussion, Other information* | | | | |
|  |  | 1 | 2 | 3 | 4 | 5 | 6 | 7 | 8 | 9 | 10 | 11 | 12 | 13 | 14 | 15 | 16 | 17 | 18 | 19 | 20 | 21 | 22 |
| Aliverini  2009 | 20 | 426 | 426 | 427 | 427 | 427 | 427 | 427 | 428 | 0 | 0 | 428 | 428 | 428 | 428-430 | 428-432 | 428-430 | 431-432 | 433 | 434 | 433-434 | 434 | 426 |
| Avouac  2011 | 19 | 476 | 476 | 476 | 476 | 476 | 476 | 476-477 | 477 | 0 | 477 | 477 | 0 | 477 | 477-480 | 477 | 477 | 0 | 478 | 480 | 478-480 | 480 | 476 |
| Avouac  2017 | 21 | 1-2 | 3 | 3 | 4-5 | 4 | 4 | 4-6 | 7 | 7 | 0 | 6-7 | 7 | 8 | 8-12 | 8-12 | 8-12 | 8-12 | 12-13 | 16 | 13-16 | 16 | 16 |
| Barnett  1988 | 10 | 121 | 121 | 0 | 0 | 0 | 0 | 0 | 0 | 0 | 0 | 0 | 0 | 0 | 122 | 122 | 122 | 123 | 123-124 | 123-124 | 123-124 | 123-124 | 0 |
| Barnett  1969 | 9 | 992 | 0 | 0 | 0 | 992 | 992 | 0 | 0 | 0 | 993 | 0 | 0 | 993 | 993-997 | 0 | 993-997 | 0 | 997 | 0 | 998-999 | 0 | 0 |
| Boonstra  2018 | 19 | 109 | 109-110 | 110 | 110 | 110 | 110 | 110-111 | 111 | 0 | 0 | 111 | 111 | 111 | 111-S112 | 111-S113 | 111-S114 | 114-S115 | 116 | 116 | 116 | 116 | 0 |
| Bruni  2015 | 17 | 72 | 72 | 72 | 73 | 72 | 73 | 73 | 73 | 0 | 0 | 0 | 0 | 73 | 73 | 73 | 73 | 0 | 73-74 | 73-74 | 73-74 | 75 | 75 |
| Caetano  2018 | 20 | 1 | 1-2 | 2 | 2 | 2 | 2 | 2 | 2 | 0 | 0 | 2 | 2 | i2 | 2-4 | 2-4 | 2-4 | 4 | 4 | 5 | 4-6 | 6 | 6 |
| Caramaschi  2007 | 21 | 1566 | 1566 | 1566 | 1566 | 1566 | 1566 | 1567 | 1567 | 0 | 1567 | 1567 | 1567 | 1567 | 1567 | 1567-1568 | 1567-1568 | 1568 | 1568 | 1568 | 1569 | 1569 | 1569 |
| Caramaschi  2015 | 5 | 0 | 0 | 0 | 0 | 0 | 0 | 0 | 0 | 0 | 0 | 0 | 0 | 0 | 0 | 2 | 2 | 0 | 2 | 2 | 2 | 0 | 0 |
| Chakravarty  2015 | 22 | 1 | 2 | 2 | 2 | 2 | 2 | 2-3 | 3 | 3 | 3 | 3 | 3 | 3 | 3 | 4-10 | 4-10 | 4-10 | 10-11 | 11 | 12 | 12 | 13 |
| Chen  1984 | 18 | 812 | 812 | 813 | 813 | 813 | 813 | 813-814 | 813-814 | 0 | 0 | 814 | 814 | 815 | 815-817 | 815-817 | 815-817 | 818-821 | 821 | 0 | 821-822 | 822 | 0 |
| Coppo  2013 | 19 | 868 | 868 | 868-867 | 867 | 867 | 867 | 867 | 867 | 0 | 867 | 867 | 867 | 867 | 867-868 | 867-868 | 867-868 | 867-868 | 868 | 868 | 868 | 0 | 0 |
| Cutolo  2004 | 19 | 719 | 719 | 720 | 720 | 720 | 720 | 720 | 720 | 0 | 0 | 720 | 720 | 721 | 722 | 722 | 722 | 723 | 724 | 725 | 724-725 | 725 | 0 |
| Cutolo  2016 | 22 | 2527-2528 | 2528 | 2528 | 2528 | 2528 | 2528 | 2528 | 2529 | 2529 | 2529 | 2529-2533 | 2529-2533 | 2533 | 2529-2535 | 2529-2535 | 2529-2535 | 2529-2535 | 2535 | 2535 | 2536 | 2537 | 2537 |
| Falkner  2000 | 19 | 1196 | 1196 | 1197 | 1197 | 1197 | 1197 | 1197 | 0 | 0 | 0 | 1197 | 1198 | 1198 | 1198-1200 | 1198-1200 | 1198-1200 | 1198-1200 | 1200 | 1201 | 1201 | 1201 | 1196 |
| Ferri  1991 | 18 | 1826 | 1826 | 1826 | 1826 | 1827 | 1827 | 1827 | 0 | 0 | 0 | 1827 | 1827 | 1827 | 1827-1830 | 1827-1830 | 1827-1830 | 1827-1830 | 1830 | 1831 | 1830-1831 | 1831 | 0 |
| Ferri  2002 | 17 | 0 | 139 | 140 | 141 | 140 | 140 | 142 | 0 | 0 | 0 | 143 | 143 | 143 | 144-149 | 144-149 | 144-146 | 146-149 | 149-150 | 151 | 150-151 | 152 | 0 |
| Foocharoen  2016 | 17 | 312 | 312 | 313 | 313 | 313 | 313 | 0 | 0 | 0 | 0 | 0 | 315 | 315 | 313-316 | 313-316 | 313-316 | 316 | 317 | 318 | 318 | 318 | 218 |
| Foocharoen  2017 | 20 | 1572 | 1572 | 1573 | 1573 | 1573 | 1573 | 1573 | 0 | 0 | 1574 | 1574 | 1574 | 1574 | 1574-1579 | 1574-1579 | 1574-1577 | 1578-1579 | 1575 | 1575 | 1577 | 1579 | 1580 |
| Franks  2019 | 21 | 1701 | 1702 | 1702 | 1702 | 1703 | 1703 | 1703 | 1703 | 0 | 1703 | 1703 | 1703 | 1704 | 1704-1708 | 1704-1708 | 1704-1706 | 1707-1708 | 1708-1709 | 1709 | 1709 | 1709 | 1701 |
| Frost  2019 | 15 | 933 | 933 | 934 | 934 | 934 | 0 | 934 | 0 | 0 | 0 | 934 | 934 | 0 | 0 | 935 | 935 | 936 | 937 | 938 | 938 | 938 | 0 |
| Giordano  1986 | n/a |  |  |  |  |  |  |  |  |  |  |  |  |  |  |  |  |  |  |  |  |  |  |
| Gliddon  2011 | 15 | 702 | 0 | 0 | 702 | 702 | 702 | 0 | 0 | 0 | 0 | 0 | 702 | 702 | 702-703 | 703 | 703 | 703 | 704 | 704 | 704 | 704 | 704 |
| Goetz  1945 | 0 | 0 | 0 | 0 | 0 | 0 | 0 | 0 | 0 | 0 | 0 | 0 | 0 | 0 | 0 | 0 | 0 | 0 | 0 | 0 | 0 | 0 | 0 |
| Gordon  2018 | 21 | 1 | 2 | 2 | 3 | 3 | 3 | 3 | 3 | 0 | 3 | 4 | 4 | 5 | 6 | 6 | 6 to 7 | 7 to 8 | 8 | 8 | 8 to 9 | 9 | 9 |
| Graf  2012 | 17 | 102 | 102-103 | 103 | 104 | 104 | 104 | 104 | 0 | 0 | 0 | 0 | 0 | 104 | 104 | 104-105 | 104-105 | 105 | 105-108 | 108 | 107-108 | 108 | 108 |
| Greidinger  2001 | 20 | 796 | 796 | 796 | 797 | 797 | 797 | 797 | 797 | 0 | 798 | 798 | 798 | 798 | 798 | 799 | 799 | 799 | 799 | 800 | 800 | 800 | 0 |
| Haddon  2017 | 21 | 631 | 631-632 | 632 | 632 | 632 | 632 | 633 | 633 | 0 | 633 | 633 | 633 | 633 | 633-634 | 634 | 634-635 | 635-636 | 636 | 636 | 636 | 636 | 631 |
| Hamaguchi  2008 | 20 | 487 | 487 | 488 | 488 | 488 | 488 | 488-489 | 489 | 0 | 488 | 489 | 489 | 489-492 | 489-492 | 489-492 | 489-492 | 489-492 | 492 | 493 | 493 | 494 | 0 |
| Hamaguchi  2015 | 20 | 1045 | 1045 | 1046 | 1046 | 1046 | 1046 | 1046 | 0 | 0 | 1046 | 1046 | 1046 | 1046 | 1046 | 1046-1049 | 1046-1049 | 1046-1049 | 1049 | 1050 | 1050 | 1050 | 1045 |
| Hanke  2010 | 19 | 2548 | 2548 | 2548 | 2549 | 2549 | 2548 | 2549 | 0 | 0 | 0 | 2549 | 2549 | 2549 | 2549 | 2549-2551 | 2549-2551 | 2549-2551 | 2551 | 2551 | 2552 | 2552 | 2548 |
| Harvey  1999 | 19 | 345 | 345 | 346 | 346 | 346 | 346 | 346 | 346 | 0 | 0 | 0 | 347 | 397 | 397 | 397-399 | 397-399 | 397-399 | 400 | 400 | 400 | 401 | 401 |
| Herrick  2004 | 18 | 1776 | 1776 | 1777 | 1777 | 1777 | 1777 | 1777 | 0 | 0 | 0 | 1777 | 1777 | 1778 | 1778 | 1778-1779 | 1778-1779 | 0 | 1779 | 1780 | 1780 | 1780 | 1781 |
| Hesselstrand  2003 | 19 | 534 | 534 | 535 | 535 | 535 | 535 | 535 | 0 | 0 | 0 | 535 | 536 | 536 | 536 | 536 | 537 | 537 | 537 | 538 | 539 | 539 | 539 |
| Hinchcliff  2013 | 18 | 1797 | 1780 | 1780 | 1788 | 1788 | 1788 | 1788 | 0 | 0 | 0 | 0 | 1789 | 1781 | 1781 | 1782 | 1782 | 1783 | 1784 | 1784 | 1785 | 1784 | 1789 |
| Hoa  2016 | 20 | 1 | 2 | 2 | 3 | 3 | -4 | 4 | 4 | 0 | 0 | 3-4 | 4 | 4 | 4 | 4-5 | 4 | 5 | 6 | 9 | 7-8 | 9 | 10 |
| Hofstee  2009 | 18 | 191 | 191 | 191 | 192 | 192 | 192 | 192 | 0 | 0 | 192 | 0 | 192 | 192 | 192-193 | 192 | 193 | 193 | 194 | 194 | 194-195 | 195 | 0 |
| Holzmann  1987 | 0 | 0 | 0 | 0 | 0 | 0 | 0 | 0 | 0 | 0 | 0 | 0 | 0 | 0 | 0 | 0 | 0 | 0 | 0 | 0 | 0 | 0 | 0 |
| Houtman  1985 | 16 | 603 | 603 | 603 | 604 | 604 | 604 | 0 | 0 | 0 | 0 | 0 | 605 | 605 | 605-606 | 606 | 607 | 607 | 608 | 608 | 608 | 609 | 0 |
| Hudson  2007 | 18 | 754 | 754 | 754 | 755 | 755 | 755 | 755 | 755 | 0 | 0 | 755 | 755 | 755-756 | 755-756 | 0 | 755-756 | 0 | 756 | 756 | 756 | 756 | 756 |
| Hudson  2012 | 22 | 787 | 787 | 788 | 788 | 788 | 788 | 788 | 788 | 788 | 788 | 788 | 788 | 789 | 790 | 790 | 790 | 791 | 792 | 793 | 792 | 793 | 793 |
| Igusa  2018 | 19 | 1 | 2 | 2 | 2 | 2 | 2 | 2 | 2 | 0 | 0 | 0 | 3 | 3 | 3,4 | 3,4, | 3,4 | 5 | 5,6 | 6 | 5,6 | 6,7,8 | 8 |
| Iniesta Arandia  2017 | 19 | 98 | 98 | 99 | 99 | 99 | 99 | 99 | 0 | 0 | 0 | 100 | 100 | 100 | 100 | 100-102 | 100 | 101 | 103 | 103 | 103 | 103 | 104 |
| Ingegnoli  2013 | 21 | 122-123 | 123 | 123 | 123 | 123 | 123 | 123 | 123 | 0 | 124 | 123 | 123 | 125 | 125 | 125-126 | 125 | 126 | 126 | 127 | 126-127 | 127 | 127-128 |
| Kenik  1981 | 14 | 885 | 885 | 885 | 886 | 886 | 886 | 886 | 886-889 | 0 | 0 | 0 | 0 | 890 | 890 | 0 | 890 | 0 | 890 | 0 | 890 | 891 | 0 |
| Kuwana  2005 | 20 | 2425 | 2425 | 2426 | 2426 | 2426 | 2426 | 2426 | 2426 | 0 | 0 | 2426-2427 | 2427 | 2427 | 2427 | 2427 | 2427-2430 | 2428-2431 | 2431 | 2431 | 2432 | 2432 | 2425 |
| Lefford  1986 | 16 | 741 | 741 | 741 | 741-742 | 642 | 642 | 742 | 0 | 0 | 0 | 0 | 743 | 743-744 | 744-746 | 744-746 | 744-745 | 745-747 | 747-749 | 0 | 749 | 749 | 0 |
| LeRoy  1988 | 4 | 202 | 0 | 0 | 0 | 0 | 0 | 0 | 0 | 0 | 0 | 0 | 0 | 0 | 0 | 0 | 0 | 0 | 204 | 0 | 204 | 204 | 0 |
| LeRoy  2001 | 5 | 1573 | 1573 | 0 | 0 | 0 | 0 | 0 | 0 | 0 | 0 | 0 | 0 | 0 | 0 | 0 | 0 | 0 | 1573-1575 | 0 | 1574-1575 | 1574-1575 | 0 |
| Liaskos  2017 | 19 | 414 | 414 | 414 | 415 | 415 | 416 | 416 | 416 | 0 | 0 | 415-416 | 416 | 417 | 416-417 | 417 | 416-417 | 0 | 417-418 | 419 | 418-419 | 419 | 419 |
| Lonzetti  2001 | 7 | 735 | 735 | 735 | 0 | 0 | 0 | 0 | 0 | 0 | 0 | 0 | 0 | 0 | 0 | 0 | 0 | 0 | 736 | 736 | 736 | 736 | 0 |
| Lovy  1985 | 15 | 496 | 496 | 496 | 496 | 497 | 497 | 496-497 | 0 | 0 | 0 | 0 | 0 | 499 | 500 | 500 | 500 | 0 | 500 | 500 | 500 | 500 | 0 |
| Mahoney  2015 | 22 | 1 | 1 to 2 | 3 | 16 | 16 | 16 | 17 | 17-18 | 17 | 17 | 17 | 17 | 4 | 4 | 4 | 4 to 6 | 7 to 13 | 13 | 15 | 16 | 16 | 1 |
| Manfredi  2015 | 17 | 133 | 134 | 134 | 134 | 135 | 135 | 135 | 135-136 | 0 | 0 | 136 | 136 | 136 | 137-138 | 138 | 137-139 | 0 | 139 | 0 | 139 | 140 | 0 |
| Maricq  2004 | 18 | 5 | 6 | 6 | 7 | 7 | 8 | 8 | 8 | 0 | 0 | 0 | 8 | 8 | 8 to 9 | 8 to 11 | 8 to 10 | 11 | 11 to 12 | 12 | 12 | 12 | 0 |
| Martyanov  2017 | 20 | 1 to 2 | 2 | 3 | 4 | 4 | 4 | 4 | 3 to 4 | 0 | 0 | 4 | 5 | 5 | 5 | 5 to 6 | 6 | 6 to 11 | 11 to 14 | 14 | 11 to 14 | 14 | 1 |
| Masi  1988 | 6 | 894 | 894 | 894 | 0 | 0 | 0 | 0 | 0 | 0 | 0 | 0 | 0 | 0 | 0 | 0 | 0 | 0 | 894-896 | 0 | 894-896 | 894-896 | 0 |
| McCarthy  1983 | 17 | 1 | 1 | 2 | 2 | 2 | 2 | 2 | 2 | 0 | 0 | 0 | 0 | 3 | 3 | 3 to 4 | 3 to 4 | 4 to 5 | 5 | 6 | 6 | 6 | 0 |
| Milano  2008 | 21 | 1 | 1 | 2 | 15 | 15 | 15 | 15 | 16 | 0 | 16 | 17 | 16 | 2 | 2 to 3 | 2 to 3 | 2 to 4 | 5 to 13 | 13 | 14 | 13 to 15 | 15 | 1 |
| Ostojic  2006 | 16 | 453 | 454 | 454 | 454 | 454 | 455 | 455 | 0 | 0 | 0 | 0 | 455 | 455 | 456 | 456 | 456 | 0 | 457 | 457 | 457 | 457 | 0 |
| Patterson  2015 | 18 | 3234 | 3235 | 2325 | 2325 | 2325 | 2325 | 2325 | 2325 | 0 | 0 | 0 | 2326 | 2326 | 2326 | 2326 | 2326-2327 | 2327-2340 | 2340 | 2341 | 2340-2342 | 2342 | 0 |
| Pendergrass  2012 | 17 | 1 | 1 to 2 | 2 | 8 | 8 | 8 | 0 | 0 | 0 | 0 | 0 | 8 | 2 | 2 to 3 | 2 to 4 | 2 to 4 | 4 to 6 | 6 | 7 | 6 to 7 | 8 | 8 |
| Perosa  2013 | 21 | 1 | 2 | 2 | 2 | 2 | 3 | 3 | 3 | 3 | 3 | 3 | 3 to 4 | 4 | 4 | 5 to 7 | 5 to 6 | 6 to 8 | 8 | 9 | 9 | 9 | 0 |
| Rodnan  1979 | 6 | 5 | 5 | 5 | 0 | 0 | 0 | 0 | 0 | 0 | 0 | 0 | 0 | 0 | 0 | 0 | 0 | 0 | 5 to 11 | 0 | 5 to 11 | 10 to 11 | 0 |
| Salazar  2015 | 19 | 1 to 2 | 3 | 3 | 3 | 3 | 3 | 4 to 5 | 5 | 0 | 0 | 4 to 5 | 5 | 6 | 6 | 7 | 7 | 0 | 7 | 8 | 8 to 10 | 11 | 11 |
| Sambataro  2014 | 19 | 1 | 1 | 2 | 2 | 2 | 2 | 2 | 0 | 0 | 0 | 3 | 3 | 3 | 3 to 4 | 3 to 4 | 4 | 5 to 6 | 6 | 6 to 7 | 6 to 7 | 7 | 7 |
| Sanchez-Montalva  2014 | 19 | 177 | 177 | 178 | 178 | 178 | 178 | 179 | 179 | 0 | 0 | 178-179 | 179 | 179 | 179 | 179-180 | 719 | 180 | 180 | 181 | 181 | 181-182 | 0 |
| Santiago  2007 | 19 | 1528 | 1528 | 1529 | 1529 | 1529 | 1529 | 1529 | 0 | 0 | 1529 | 0 | 1529 | 1529 | 1529 | 1530 | 1530-1531 | 1532 | 1532 | 1533 | 1532-1533 | 1533 | 1528 |
| Sato  1998 | 16 | 2135 | 2135 | 2135 | 2136 | 2136 | 2136 | 0 | 0 | 0 | 0 | 0 | 2136 | 2136 | 2136 | 2136-2138 | 2136-2138 | 2137-2138 | 2138 | 2138 | 2138 | 2138 | 0 |
| Sato  2009 | 20 | 219 | 220 | 220 | 221 | 221 | 220 | 221 | 221 | 0 | 0 | 221 | 221-222 | 222 | 222 | 222-223 | 222-223 | 223-224 | 224 | 226 | 225 | 226 | 226 |
| Satoh  2009 | 18 | 1570 | 1570 | 1570 | 1570-1571 | 1571 | 1571 | 1571 | 0 | 0 | 0 | 0 | 1571 | 1571 | 1572 | 1572 | 1572 | 1572-1573 | 1573 | 1573 | 1573 | 1574 | 1574 |
| Scussel-Lonzetti  2002 | 18 | 0 | 154 | 154 | 154 | 155 | 155 | 155 | 155 | 0 | 0 | 156 | 156 | 156 | 157 | 157 | 157-158 | 159-162 | 162 | 165 | 163-164 | 165-166 | 0 |
| Sebastiani  2009 | 16 | 688 | 688 | 688 | 688-689 | 689 | 689 | 689 | 0 | 0 | 0 | 0 | 0 | 690 | 691 | 691 | 691 | 692 | 629 | 693 | 692-693 | 693 | 0 |
| Sebastiani  2012 | 15 | 67 | 67 | 67 | 67 | 67 | 67 | 67 | 0 | 0 | 0 | 0 | 0 | 68 | 68 | 68 | 68 | 68-69 | 69 | 0 | 69 | 69 | 0 |
| Sebastiani  2013 | n/a |  |  |  |  |  |  |  |  |  |  |  |  |  |  |  |  |  |  |  |  |  |  |
| Shah  2010 | 19 | 2787 | 2787 | 2788 | 2788 | 2788 | 2788 | 2788 | 2788 | 0 | 0 | 0 | 2789 | 2789 | 2789 | 2789 | 2789-2790 | 2790-2791 | 2791 | 2792 | 2791-2793 | 2794 | 2794 |
| Shah  2019 | 18 | 1571 | 1571 | 1571 | 1572 | 1572 | 1572 | 1573-1574 | 0 | 0 | 0 | 0 | 1574 | 1575 | 1575 | 1575 | 1576 | 1577 | 1577 | 1578 | 1578 | 1578 | 1578 |
| Shayakhmetova2019 | 18 | 723 | 723 | 723 | 724 | 724 | 725 | 725 | 725 | 0 | 0 | 0 | 725 | 726 | 726 | 726 | 727 | 727 | 727 | 727 | 728 | 728 | 0 |
| Shenavandeh  2017 | n/a |  |  |  |  |  |  |  |  |  |  |  |  |  |  |  |  |  |  |  |  |  |  |
| Simeon  1997 | 19 | 723 | 723 | 723-724 | 724 | 724 | 724 | 724-725 | 724 | 0 | 0 | 725 | 725 | 725 | 725 | 726 | 726 | 726-727 | 727 | 728 | 727-728 | 728 | 0 |
| Simon  2009 | 19 | 415 | 415 | 416 | 416 | 416 | 416 | 416 | 416 | 0 | 0 | 0 | 417 | 417 | 417 | 418 | 418 | 419 | 420 | 421 | 420 | 421 | 421 |
| Smith  2012 | 18 | 1636 | 1636 | 1636 | 1636 | 1637 | 0 | 1637 | 1637 | 0 | 0 | 1637 | 1637 | 1637 | 1637-1637 | 1638 | 1638 | 1638 | 1638 | 0 | 1638 | 1639 | 1639 |
| Smith  2013 | 17 | 2023 | 2023 | 2023 | 2024 | 2024 | 2024 | 2024 | 0 | 0 | 0 | 2024 | 2024-2025 | 2025 | 2025 | 2025 | 2025 | 0 | 2026 | 0 | 2026 | 2026 | 2023 |
| Sobanski  2019 | 19 | 1553 | 1554 | 1554 | 1554 | 1554 | 1554 | 1554 | 0 | 0 | 0 | 1554 | 1554-1559 | 1559 | 1559-1561 | 1559-1561 | 1559-1561 | 1561-1563 | 1564-1565 | 1566 | 1566-1567 | 1567 | 1553 |
| Song  2013 | 18 | 1 | 2 | 2 | 3 | 3 | 3 | 3- 4 | 4 | 0 | 0 | 0 | 4 | 4 to 5 | 5 | 5 | 5 | 6 | 6 | 0 | 6 | 6 | 7 |
| Sulli  2013 | 15 | 634 | 634 | 634 | 635 | 635 | 0 | 0 | 0 | 0 | 0 | 635 | 635 | 635 | 635 | 636 | 635-636 | 0 | 638 | 638 | 638 | 639 | 0 |
| Taroni  2015 | 21 | 1 | 2 | 2 | 2 | 2 | 2 | 3 | 3 | 3 | 0 | 3 | 3 | 4 | 4 | 4 | 4 to 8 | 9 to 10 | 10 to 11 | 11 | 11 | 11 | 13 |
| Taroni  2017 | 16 | 1033 | 1033 | 1033 | 1040 | 1040 | 0 | 0 | 0 | 0 | 0 | 0 | 1040 | 1034 | 1034 | 1034-1035 | 1035-1036 | 1036-1038 | 1039 | 1039 | 1039-1040 | 1040 | 1040 |
| Terras  2016 | 16 | 882 | 882 | 882 | 882 | 0 | 882 | 0 | 0 | 0 | 0 | 882 | 882 | 882 | 882 | 882 | 882-883 | 0 | 884 | 884 | 884 | 884 | 882 |
| Tuffanelli  1962 | 9 | 198 | 198 | 198 | 198 | 198 | 198 | 0 | 0 | 0 | 0 | 0 | 0 | 0 | 0 | 0 | 0 | 0 | 202 | 0 | 202-203 | 202 | 0 |
| van der Kroef  2020 | 19 | 119 | 120 | 120 | 127 | 127 | 127 | 128 | 128 | 0 | 0 | 0 | 128 | 120 | 120 | 121 | 121-124 | 124 | 124-125 | 127 | 124-127 | 127 | 128 |
| Vayssairat  1992 | 18 | 356 | 356 | 356 | 357 | 357 | 357 | 357 | 357 | 0 | 0 | 357 | 357 | 358 |  | 358 | 358-359 | 359 | 360 | 0 | 361 | 361 | 0 |
| Vazquez-Abad  1994 | 16 | 248 | 248 | 248 | 248 | 248 | 249 | 249 | 0 | 0 | 0 | 0 | 249 | 249 | 249-250 | 0 | 250 | 251 | 251 | 251-252 | 252 | 252 | 0 |
| Winterbauer  1964 | 2 | 361 | 362 | 0 | 0 | 0 | 0 | 0 | 0 | 0 | 0 | 0 | 0 | 0 | 0 | 0 | 0 | 0 | 0 | 0 | 0 | 0 | 0 |
| Wodkowski  2015 | 17 | 131 | 131 | 132 | 132 | 132 | 132 | 0 | 0 | 0 | 0 | 0 | 132 | 132 | 133 | 133 | 133 | 133 | 133 | 134 | 134 | 134 | 135 |
| Wu  2007 | 18 | 47 | 48 | 48 | 49 | 49 | 49 | 49 | 0 | 0 | 0 | 49 | 49 | 49-50 | 50 | 50 | 50 | 0 | 50 | 51 | 51 | 52 | 53 |
| Wuttge  2015 | 19 | 2100 | 2100-2101 | 2101 | 2101 | 2101 | 2101 | 2101 | 2101 | 0 | 0 | 0 | 2102 | 2102 | 2102-2103 | 2102 | 2102 | 2103-2105 | 2016 | 2106 | 2106-2107 | 2107 | 2017 |
