# Supplementary material for: Systematic Analysis of the Literature in Search of Defining Systemic Sclerosis Subsets
Source: J Rheumatol. Author manuscript; Available in PMC 2023 Oct 29. (PMC10613330; doi:10.3899/jrheum.201594)
Supplement: Appendix 2 [file NIHMS1895227-supplement-Appendix_2.docx]

Appendix II. References (continued)

101. Houtman PM, Kallenberg CG, Wouda AA, The TH. Decreased nailfold capillary density in Raynaud's phenomenon: a reflection of immunologically mediated local and systemic vascular disease? Ann Rheum Dis 1985;44:603-9.

102. Bredemeier M, Xavier RM, Capobianco KG, Restelli VG, Rohde LE, Pinotti AF, et al. Nailfold capillary microscopy can suggest pulmonary disease activity in systemic sclerosis. J Rheumatol 2004;31:286-94.

103. Ostojic P, Damjanov N. Different clinical features in patients with limited and diffuse cutaneous systemic sclerosis. Clin Rheumatol 2006;25:453-7.

104. Lefford F, Edwards JC. Nailfold capillary microscopy in connective tissue disease: a quantitative morphological analysis. Ann Rheum Dis 1986;45:741-9.

105. Lovy M, MacCarter D, Steigerwald JC. Relationship between nailfold capillary abnormalities and organ involvement in systemic sclerosis. Arthritis Rheum 1985;28:496-501.

106. Kenik JG, Maricq HR, Bole GG. Blind evaluation of the diagnostic specificity of nailfold capillary microscopy in the connective tissue diseases. Arthritis Rheum 1981;24:885-91.

107. Hofstee HM, Vonk Noordegraaf A, Voskuyl AE, Dijkmans BA, Postmus PE, Smulders YM, et al. Nailfold capillary density is associated with the presence and severity of pulmonary arterial hypertension in systemic sclerosis. Ann Rheum Dis 2009;68:191-5.

108. Greidinger EL, Gaine SP, Wise RA, Boling C, Housten-Harris T, Wigley FM. Primary pulmonary hypertension is not associated with scleroderma-like changes in nailfold capillaries. Chest 2001;120:796-800.

109. Alivernini S, De Santis M, Tolusso B, Mannocci A, Bosello SL, Peluso G, et al. Skin ulcers in systemic sclerosis: determinants of presence and predictive factors of healing. J Am Acad Dermatol 2009;60:426-35.

110. Sebastiani M, Manfredi A, Colaci M, D'Amico R, Malagoli V, Giuggioli D, et al. Capillaroscopic skin ulcer risk index: a new prognostic tool for digital skin ulcer development in systemic sclerosis patients. Arthritis Rheum 2009;61:688-94.

111. Sebastiani M, Manfredi A, Vukatana G, Moscatelli S, Riato L, Bocci M, et al. Predictive role of capillaroscopic skin ulcer risk index in systemic sclerosis: a multicentre validation study. Ann Rheum Dis 2012;71:67-70.

112. Sebastiani M, Manfredi A, Lo Monaco A, Praino E, Riccieri V, Grattagliano V, et al. Capillaroscopic Skin Ulcers Risk Index (CSURI) calculated with different videocapillaroscopy devices: how its predictive values change. Clin Exp Rheumatol 2013;31:115-7.

113. Manfredi A, Sebastiani M, Carraro V, Iudici M, Bocci M, Vukatana G, et al. Prediction risk chart for scleroderma digital ulcers: a composite predictive model based on capillaroscopic, demographic and clinico-serological parameters. Clin Hemorheol Microcirc 2015;59:133-43.

114. Avouac J, Lepri G, Smith V, Toniolo E, Hurabielle C, Vallet A, et al. Sequential nailfold videocapillaroscopy examinations have responsiveness to detect organ progression in systemic sclerosis. Semin Arthritis Rheum 2017;47:86-94.

115. Lonzetti LS, Joyal F, Raynauld JP, Roussin A, Goulet JR, Rich E, et al. Updating the American College of Rheumatology preliminary classification criteria for systemic sclerosis: addition of severe nailfold capillaroscopy abnormalities markedly increases the sensitivity for limited scleroderma. Arthritis Rheum 2001;44:735-6.

116. Hudson M, Taillefer S, Steele R, Dunne J, Johnson SR, Jones N, et al. Improving the sensitivity of the American College of Rheumatology classification criteria for systemic sclerosis. Clin Exp Rheumatol 2007;25:754-7.

117. Herrick AL, Moore TL, Murray AK, Whidby N, Manning JB, Bhushan M, et al. Nail-fold capillary abnormalities are associated with anti-centromere antibody and severity of digital ischaemia. Rheumatology (Oxford) 2010;49:1776-82.

118. Sambataro D, Sambataro G, Zaccara E, Maglione W, Polosa R, Afeltra AM, et al. Nailfold videocapillaroscopy micro-haemorrhage and giant capillary counting as an accurate approach for a steady state definition of disease activity in systemic sclerosis. Arthritis Res Ther 2014;16:462.

119. von Elm E, Altman DG, Egger M, Pocock SJ, Gotzsche PC, Vandenbroucke JP, et al. The Strengthening the Reporting of Observational Studies in Epidemiology (STROBE) statement: guidelines for reporting observational studies. Lancet 2007;370:1453-7.

120. Clements PJ, Hurwitz EL, Wong WK, Seibold JR, Mayes M, White B, et al. Skin thickness score as a predictor and correlate of outcome in systemic sclerosis: high-dose versus low-dose penicillamine trial. Arthritis Rheum 2000;43:2445-54.

121. Hanitsch LG, Burmester GR, Witt C, Hunzelmann N, Genth E, Krieg T, et al. Skin sclerosis is only of limited value to identify SSc patients with severe manifestations--an analysis of a distinct patient subgroup of the German Systemic Sclerosis Network (DNSS) Register. Rheumatology (Oxford) 2009;48:70-3.

122. Mak AC, Tang PL, Cleveland C, Smith MH, Kari Connolly M, Katsumoto TR, et al. Brief Report: Whole-Exome Sequencing for Identification of Potential Causal Variants for Diffuse Cutaneous Systemic Sclerosis. Arthritis Rheumatol 2016;68:2257-62.

123. Whitfield ML, Finlay DR, Murray JI, Troyanskaya OG, Chi JT, Pergamenschikov A, et al. Systemic and cell type-specific gene expression patterns in scleroderma skin. Proc Natl Acad Sci U S A 2003;100:12319-24.

124. Taroni JN, Greene CS, Martyanov V, Wood TA, Christmann RB, Farber HW, et al. A novel multi-network approach reveals tissue-specific cellular modulators of fibrosis in systemic sclerosis. Genome Med 2017;9:27.

125. Lima H MC, Paiva E. . A study of systemic scleroderma in the southern region of Brazil: Cutaneous manifestations and clinical and laboratory marker association. J Am Acad Dermatol 60:AB63.

126. Wodkowski M, Hudson M, Proudman S, Walker J, Stevens W, Nikpour M, et al. Clinical correlates of monospecific anti-PM75 and anti-PM100 antibodies in a tri-nation cohort of 1574 systemic sclerosis subjects. Autoimmunity 2015;48:542-51.

127. Maes L, Blockmans D, Verschueren P, Westhovens R, De Beeck KO, Vermeersch P, et al. Anti-PM/Scl-100 and anti-RNA-polymerase III antibodies in scleroderma. Clin Chim Acta 2010;411:965-71.

128. Villalta D, Imbastaro T, Di Giovanni S, Lauriti C, Gabini M, Turi MC, et al. Diagnostic accuracy and predictive value of extended autoantibody profile in systemic sclerosis. Autoimmun Rev 2012;12:114-20.

129. Maricq HR, Harper FE, Khan MM, Tan EM, LeRoy EC. Microvascular abnormalities as possible predictors of disease subsets in Raynaud phenomenon and early connective tissue disease. Clin Exp Rheumatol 1983;1:195-205.

130. Cutolo M, Sulli A, Pizzorni C, Accardo S. Nailfold videocapillaroscopy assessment of microvascular damage in systemic sclerosis. J Rheumatol 2000;27:155-60.

131. Markusse IM, Meijs J, de Boer B, Bakker JA, Schippers HPC, Schouffoer AA, et al. Predicting cardiopulmonary involvement in patients with systemic sclerosis: complementary value of nailfold videocapillaroscopy patterns and disease-specific autoantibodies. Rheumatology (Oxford) 2017;56:1081-1088.
